# Spatiotemporal feature learning for actin dynamics

**DOI:** 10.1371/journal.pone.0318036

**Published:** 2025-03-05

**Authors:** Siddhartha Saha, Qixin Yang, Wolfgang Losert, Alexandre V Morozov, Anirvan M Sengupta

**Affiliations:** 1 Department of Physics and Astronomy, Rutgers University, Piscataway, New Jersey, United States of America; 2 Department of Physics, University of Maryland College Park, College Park, Maryland, United States of America; 3 Center for Quantitative Biology, Rutgers University, Piscataway, New Jersey, United States of America; 4 Center for Computational Mathematics, Flatiron Institute, New York, New York, United States of America; 5 Center for Computational Quantum Physics, Flatiron Institute, New York, New York, United States of America; KIST: Korea Institute of Science and Technology,KOREA/GERMANY

## Abstract

The social amoeba *Dictyostelium discoideum* is a standard model system for studying cell motility and formation of biological patterns. *D. discoideum* cells form protrusions and migrate via cytoskeletal reorganization driven by coordinated waves of actin polymerization and depolymerization. Assembly and disassembly of actin filaments are regulated by a complex network of biochemical reactions, exhibiting sensitivity to external physical cues such as stiffness, composition and surface topography of the extracellular matrix, as well as the presence of external electric fields. In this study, we investigate whether the cellular microenvironment, and in particular the presence of electric fields and the nano-topography type, can be directly inferred from images or videos of actin waves. We employ three machine learning techniques to analyze the resulting videos: dictionary learning, scattering transforms, and optical flow. We predict the type of the extracellular environment by observing actin waves frame-by-frame and identifying key visual features that help classify cell motion by the microenvironment type. Our analysis reveals that the decomposition of static images into an adaptive basis of visual primitives provides a robust approach to classifying cells by the nano-topography type. In contrast, predicting whether cells are moving under the influence of an external electric field requires tracking of stable cellular features such as corners and edges over a period of time. We expect our computational approach to be useful in many settings where non-trivial collective dynamics is observed with the help of fluorescent labeling and video microscopy.

## Introduction

Directed cell migration plays a key role in such diverse biological processes as embryonic development [1], neural development [2], cancer metastasis [3], wound healing [4], immune response [5,6], and angiogenesis [7]. Directed cellular motion, division and differentiation are enabled by cytoskeletal dynamics and in particular by actin dynamics [8,9]. Actin waves resulting from directional polymerization and depolymerization of actin filaments act as an important driver of cell migration and polarization [10,11]. Extracellular microenvironment is a key modulator of actin dynamics: substrate stiffness [12], biochemical composition [13], and surface topography [14] are all known to influence cell dynamics.

In particular, cells adjust their migration to the local shape of the surface on which they move, in a process known as contact guidance [15]. Nano-engineered surfaces, which provide a controlled extracellular environment, have been extensively used to investigate the effects of surface shape and topography on the patterns of cell motion [16–21]. Overall, these studies report profound changes in both cell center-of-mass motion and cell shape dynamics when cells are in contact with custom-designed nano-topographic surfaces. Another important physical factor influencing cell behavior and migration patterns is the electric field. Electrotaxis – directed migration of cells under the guidance of an electric field – is important in such diverse biological processes as development, wound healing, and tissue regeneration [22–25].

Here we study how cell motion and the corresponding actin polymerization and depolymerization waves are affected by both extracellular microenvironment (modeled using nano-topographic surfaces) and extracellular stimuli (modeled by applying electric field to the cells). We employ the social amoeba *Dictyostelium discoideum* as a model organism. *D. discoideum* is commonly accepted as a model system for amoeboid motility [26,27]. In order to better distinguish between actin wave response to electric fields and the overall cell motion, we use electro-fused giant *D. discoideum* cells whose diameters are up to 10 times larger than those in a normal, unfused cell. Indeed, electrofusion merges tens of *D. discoideum* cells into a single cell with the correspondingly larger volume and surface contact area [28]. Such giant cells provide an unparalleled opportunity for studying actin wave dynamics in detail [29,30]. We use video microscopy to track *D. discoideum* cells and observe the time development of actin polymerization waves.

Previous work by some of the authors has established that actin polymerization dynamics is profoundly affected by both surface nano-topography and the presence of electric fields [19–21,29,30]. Over the last decades, teaching computers to recognize phenotypes from microscopic images has become an important problem in both computer vision and quantitative biology [31–33]. In particular, in the context of phenotyping cellular motility and morphodynamics, extraction of relevant features has become a critical issue [34]. One way to extract relevant features is to set up supervised learning problems and ascertain the predictive power of various extracted features.

More specifically, one of the main goals of this work is to introduce feature learning, also called representation learning [35], in the context of biological image processing. Whereas many method development papers in biophysics are motivated by particular applications, we want to demonstrate that our representations are generally informative and, potentially, of wide utility to the biophysics community. One way to think about representations or features is in terms of the compression of the original data, which throws away some aspects of the data but retains the information relevant to a particular task [36]. If the focus is on particular biological tasks, a very lossy compression scheme may prove quite useful. For example, many handcrafted features, such as granularity or texture in CellProfiler [37], are extremely lossy in the sense of image reconstruction and yet are useful for tasks like image segmentation. On the other hand, one might not know in advance what tasks (e.g., tracking subtle phenotypic distinctions) one ultimately wants to tackle. In that case, it makes sense to control the image reconstruction loss. Dictionary learning, and sparse auto-encoders in general [35], take this approach. The image reconstruction itself is not the ultimate goal, but a way to ensure that the representation is a low-loss compression. The compression makes it easier to train small or regularized models that distinguish between different cellular phenotypes. 

In this study we ask whether the cellular microenvironment can be inferred from observations of either snapshots of polymerized actin or observations of how actin waves change with time. This is a challenging task since it is diﬃcult to pinpoint which actin wave features are most responsible for the observed differences in cell behavior. We use machine learning methods borrowed from computer vision to find both static visual features in individual video frames and time-dependent patterns of actin dynamics in entire video sequences that explain the dependence of cell motility on extracellular environment or stimuli. These static or dynamic features are subsequently used to classify cells by their microenvironment type. We pay particular attention to the interpretability and explainability of the features we identify [38], since our ultimate goal is understanding the physical mechanisms of actin wave dynamics.

Specifically, we use two image analysis techniques to extract key features from individual video frames of giant *D. discoideum* cells. One is sparse dictionary learning, which is typically used to learn a latent set of features (called dictionary atoms) using a dataset of images as input. These features, common to all images in the dataset, can be subsequently employed to reconstruct the de-noised representation of each image [39–42]. The other technique, called a scattering transform, is based on a multi-resolution wavelet analysis of the image frames [43–46]. In contrast to sparse dictionary learning and deep convolutional neural networks [47,48], scattering transforms employ a fixed set of multi-resolution features. Such features may be more easily interpretable than the latent features obtained via dictionary learning methods.

In order to investigate how actin waves change with time, we employ optical flow – a method originally developed to extract velocity vectors from movies of everyday objects [49,50] and previously employed by us to track cellular motion [21,30]. Here, we expect optical flow techniques to be useful in identifying the presence of the electric field, since turning on the field results in visibly distinct patterns of cell motion [29]. Instead of tracking images pixel-by-pixel, we focus first on extracting a limited set of key features such as corners and edges, followed by their tracking with time [51–53]. This procedure results in a more robust set of velocity flow vectors that are more amenable to subsequent analysis.

Each of the three image analysis techniques (dictionary learning, scattering transforms, optical flow) yields a compact set of statistics which represent the original image frame or video sequence. We use these summary statistics to construct feature vectors that serve as inputs to a Support Vector Machine (SVM) binary classifier [54,55]. SVMs allow us to classify image frames or entire videos by the type of surface nano-topography that the cells are moving on, and by the presence or absence of the external electric field. Successful classification indicates that our machine learning techniques have yielded a useful set of features that can be used to predict the type of extracellular environment or stimulus just by observing moving cells. Indeed, we find that both dictionary learning and scattering transforms can be used to distinguish the cells moving on flat vs. ridged surfaces, although the latter approach is less accurate when the orientation of the nano-ridges varies in the microscopy data. Furthermore, optical flow techniques are crucial in predicting whether the cell motion is influenced by the external electric field. Altogether, our algorithms establish a novel quantitative approach to analyzing microphotography and video microscopy data in various biological settings.

## Results

Our overall goal is to classify the images and the videos of *Dictyostelium* cells as a function of the surface nano-topography (flat or ridged) and the presence or absence of the electric field (see S1 and S2 Figs for representative cell images). In response to these environmental conditions, cells exhibit different patterns of actin polymerization; cell shapes or the overall patterns of cell motion may also change depending on the environment, leading to detectable differences between populations of cells observed in different conditions. Our methods also yield a compact subset of features that are most responsible for the observed differences – a challenging task given the dimensionality and the complexity of the image data.

**Table 1:** Summary of video microscopy data for moving *Dictyostelium* cells. **Env** stands for the cellular microenvironment: flat surfaces with no electric field (${Flat}_{\text{E}=0}$), flat surfaces with electric field (${Flat}_{\text{E}}$), ridged surfaces with no electric field (${Ridged}_{\text{E}=0}$), and ridged surfaces with electric field (${Ridged}_{\text{E}}$). *N_f_* denotes the number of frames in each video or sub-video; *N_t_* is the total number of frames available for each environment type; *N_v_* shows the number of videos or sub-videos for each environment type.

Env	*N_f_*												*N_t_*	*N_v_*
${Flat}_{E=0}$	95	30	61	60	98	30	31	125	62	52	45	59	748	12
${Flat}_{E}$	65	66	55	126									312	4
${Ridged}_{E=0}$	120	31	122	62	60								395	5
${Ridged}_{E}$	181	115	131	157	101	171	84	121					1061	8

### Dictionary learning on cellular images

**Fig 1:**
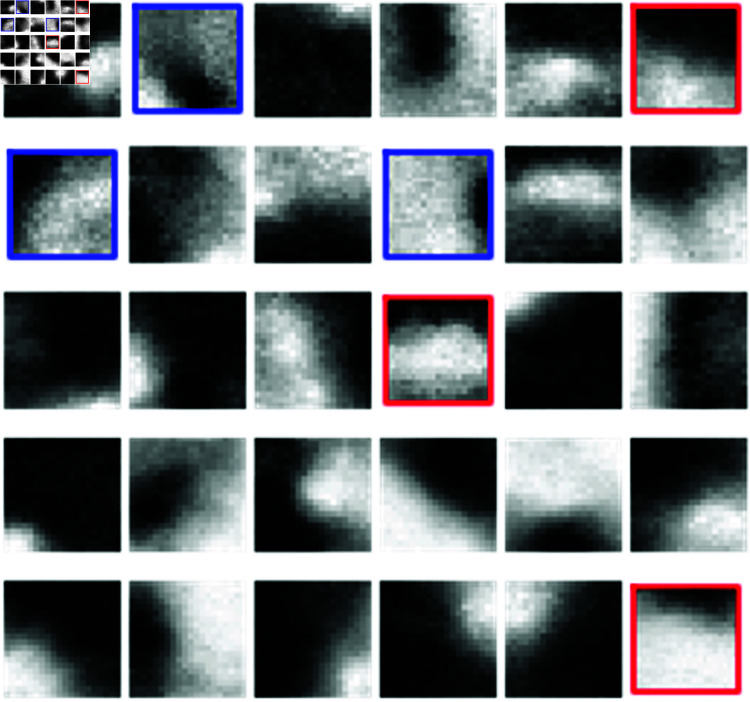
Visualization of dictionary atoms as a set of filters for sparse image reconstruction. Each dictionary atom is a 20×20 image. Highlighted are the features corresponding to top 3 positive (red squares; cells on ridged surfaces) and negative (blue squares; cells on flat surfaces) SVM weights in Fig 3B.

In this section, we use Dictionary Learning [39,41] (see Materials and Methods) to classify images of *Dictyostelium* cells as a function of the surface nano-topography (flat or ridged) and the presence or absence of the electric field. Each video is parsed into its constituent image frames and each frame is analyzed separately. Specifically, 300 randomly chosen 20×20 patches are extracted from each image; each patch may or may not overlap with the other patches that originate from the same frame. Thus, in the absence of the electric field the data matrix *X* in Eq (2) consists of N=1143×300=342900 columns of length d=20×20=400, corresponding to 748+395=1143 frames listed in Table 1. Because the matrix is so large, it is processed in batches of size 15. We choose M=100 dictionary atoms and set *λ* = 1 in Eq (3). The list of 100 predicted dictionary atoms is subsequently reduced to 30 by ranking all atoms by the reconstruction error: $\sum_{i}|x_{i}\;-\;{\left(Ds_{i}\right)}_{-j}{|}^{2}$, where the sum runs over all the patches in the dataset and ${\left(Ds_{i}\right)}_{-j}$ is given by $Ds_{i}$ in Eq (3), but with $j^{\text{th}}$ column removed from *D* and $j^{\text{th}}$ element removed from $s_{i}$ (*j* = 1*…M*). Only the atoms with 30 largest reconstruction errors are retained in the basis set (Fig 1). These images are conceptually similar to the filters learned during CNN training [47,48,54,56].

**Fig 2:**
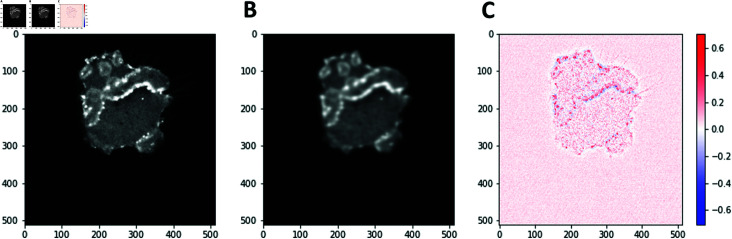
Sparse coding reconstruction of an image frame showing a single *Dictyostelium* cell. Panel (A) shows the original image, panel (B) shows the same image reconstructed using sparse coding, and panel (C) shows a colormap of the pixel-by-pixel differences between the original and the reconstructed images. The frame was extracted from a video of a *Dictyostelium* cell on the flat surface in the absence of the electric field. Note that all image frames in this study are 107.52×107.52μm in size.

In order to demonstrate that the top 30 dictionary atoms can be used to reconstruct the entire image, we extract all possible overlapping 20×20 patches from an image and represent each patch via sparse encoding in Eq (2) with M=30. The values at each pixel in the final reconstructed image are obtained by averaging over the pixel’s value in all patches that contain that pixel. The result of the reconstruction is shown in Fig 2.

Next, we construct a measure that succinctly characterizes the entire 512×512 image frame. Let us denote $x_{1},x_{2},\ldots ,x_{N}$ a set of vectorized image patches, where *N* is the total number of all overlapping 20×20 patches extracted from a given frame. Then $s_{1},s_{2},\ldots ,s_{N}$ denote the corresponding set of sparse codes for these patches. We construct a feature vector *v* such that $v_{k}=\sum_{i=1}^{N}|s_{i,k}|$ (*k* = 1*…M*), where $s_{i,k}$ denotes the $k^{\text{th}}$ component of the *M*-dimensional vector $s_{i}$. A set of 30-dimensional feature vectors computed for a suitably chosen set of frames provides an input dataset for the Support Vector Machine (SVM) binary classifier [54,57].

We use the SVM classifier implemented in scikit-learn, with the $L_{1}$ penalty on the fitting weights and C=1000. The SVM scores are defined as


$$\begin{array}{c} f(v)=w\cdot v+b, \end{array}$$ (1)

where *v* is the feature vector, *w* is the corresponding *M*-dimensional vector of weights, and *b* is the intercept [54]. Note that the components of *v* are non-negative in our case. First, we focus on differentiating between images of *Dictyostelium* cells moving on flat vs. ridged surfaces in the absence of the external electric field. In this case, the positive dataset is given by all image frames from 5 videos of cells on ridged surfaces and the negative dataset is given by all image frames from 12 videos of cells on flat surfaces (Table 1). Both positive and negative datasets are randomly divided into training and test sets in a way that prevents frames from the same video from appearing both in the training and the test set. Namely, we randomly divide the available videos into 3 subsets of  ( 2 , 1 , 2 )  videos for ridged surfaces and  ( 4 , 4 , 4 )  videos for flat surfaces. One of the 3 subsets then constitutes the test set, while the other 2 groups make up the training set. Note that this procedure results in 3 splits into test/training sets (labeled splits 1 , 2 , 3) depending on which of the 3 subsets is chosen as the test set. In the end, the test set consists of 4 videos on flat surfaces and 2 videos on ridged surfaces in split 1, 4 videos on flat surfaces and 1 video on ridged surfaces in split 2, and 4 videos on flat surfaces and 2 videos on ridged surfaces in split 3.

**Fig 3:**
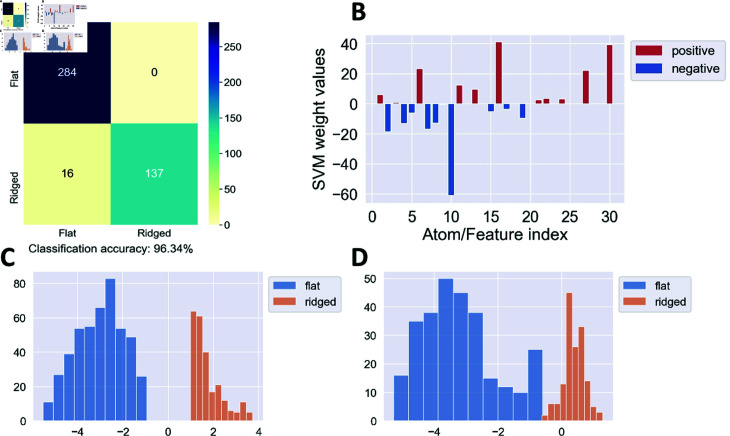
SVM classification of feature vectors obtained by dictionary learning. The features characterize images of *Dictyostelium* cells moving on flat vs. ridged surfaces in the absence of the external electric field. Shown are results for training/test set split 1. Panel (A) is the confusion matrix for the SVM classification into two nano-topography types (flat/ridged surfaces). The true labels are on the *y*-axis in all confusion matrices presented in this work. Panel (B) shows SVM weights assigned to each dictionary atom. The dictionary atoms corresponding to the top three positive and negative SVM weights are highlighted in Fig 1; the 1–30 numbering on the *x*-axis corresponds to the rows of atoms in Fig 1, starting from the upper left corner. Note that dictionary atoms characterized by positive/negative SVM weights describe features of cells on ridged/flat surfaces, respectively. Panel (C) is a histogram of SVM scores (Eq (1)) evaluated on all video frames from the training set; panel (D) is a histogram of SVM scores (Eq (1)) evaluated on all video frames from the test set.

Finally, the SVM is trained on the training set and the resulting classification accuracy on the test set is represented by the confusion matrix in Fig 3A (see S3A and S4A Figs for the classification results on the other two training/test splits; all confusion matrices shown in this work correspond to the test data unless explicitly indicated otherwise). We observe that the set of dictionary atoms shown in Fig 1 leads to high SVM classification accuracy. The top 3 dictionary atoms that contribute to differentiating images of cells on flat vs. ridged surfaces are highlighted in Fig 1. The top 3 atoms that are found more frequently in videos on ridged surfaces all appear to contain horizontal or nearly horizontal bands. In contrast, the top 3 features from videos on flat surfaces contain inclined edges and, in one case, a nearly uniformly lit square with a black vertical bar to one side. This is consistent with a previous observation that cells on nano-ridges exhibit one-dimensional actin waves guided along the horizontally-oriented ridges, in a process known as esotaxis, whereas cells on flat substrates are characterized by two-dimensional actin waves [30].

To analyze which atoms contribute the most to the classification, we plot the components of the weight vector *w* in Fig 3B. Note that positive weights correspond to the atoms that are more prominent in the image frames obtained from the videos on ridged surfaces. Likewise, negative weights correspond to the atoms that are found more readily in the image frames from the videos on flat surfaces. Finally, we show histograms of SVM scores for the training set (Fig 3C) and the test set (Fig 3D). As expected, the separation between SVM scores corresponding to the flat and ridged surfaces is higher for the training set than for the test set. However, even in the latter case the scores are well-separated, consistent with the high classification accuracy observed in Fig 3A. The corresponding results for training/test splits 2 and 3 are shown in S3B–S3D Fig and S4B–S4D Fig, respectively. We also note a high degree of reproducibility between SVM weights obtained on splits 1 and 2 (compare Fig 3B and S3B Fig), although the sets of SVM weights obtained on splits 1 and 3 are somewhat less correlated (compare Fig 3B and S4B Fig).

Classification of individual image frames into flat or ridged can be used to classify entire videos containing these frames by majority voting. In the split 1 test set, no frames depicting cells on flat surfaces were misclassified, while for the two videos of cells on ridged surfaces, 4 out of 31 frames were misclassified in one video and 12 out of 122 frames were misclassified in the other (Fig 3A). Thus, for split 1 the accuracy of video classification is 100% by majority voting. Similarly, the split 2 test set produces 100% accurate classification of each frame and therefore of entire videos (S3A Fig). In the split 3 test set, 26 out of 60 frames were misclassified for one of the two videos of cells on ridged surfaces and all other frames were classified correctly, again yielding 100% accurate video classification. Visual inspection revealed that the video with 26∕60 misclassified frames appears to have fewer horizontal features that are characteristic of cells on ridged surfaces. Despite this, the overall video accuracy classification by majority voting is 100% on all test sets.

Since the nano-ridges are oriented horizontally in all image frames (Materials and Methods), it is conceivable that our classification is aided by the advance knowledge that the ridge orientation is fixed in one direction, which would not be the case in more realistic microcellular environments. To investigate this issue, we have created an enlarged dataset in which the original images were combined with the same images rotated counterclockwise by $90^{\circ }$, $180^{\circ }$, and $270^{\circ }$. The dataset created in this way contains both horizontally and vertically oriented ridges, potentially making the classification a more challenging task. However, we do not observe a significant loss of accuracy on this combined dataset, indicating that dictionary learning is suﬃciently flexible to accommodate the loss of knowledge of the nano-ridge orientation (S5 Fig). Note that the most informative features for differentiating between flat and ridged surfaces have changed (cf. red and blue boxes in Fig 1 and S5C, S5F, S5I Fig), consistent with the fact that dictionary learning produces a set of adaptive, data-dependent visual features. The new features tend to be more oblique than before, probably to account for the mixture of vertical and horizontal nano-ridge orientations.

Next, we divided all videos in Table 1 into two groups: 15 videos on flat surfaces and 9 videos on ridged surfaces, regardless of the presence or absence of the external electric field. These videos were randomly split into 3 subgroups of  ( 5 , 5 , 5 )  videos for flat surfaces and  ( 3 , 3 , 3 )  videos for ridged surfaces, again ensuring that no frames from the same video appear in both the training and the test sets. The subgroups of videos were used to construct 3 equivalent training/test splits as before, with one subgroup in the test set and the other two in the training set. The SVM classifier was trained on these data as described above, using the same scikit-learn implementation and the same parameter settings.

The resulting confusion matrix indicates a significant loss of accuracy when the videos of cells moving under the influence of the external electric field are included into the dataset (S6 Fig). The lack of accuracy is even more pronounced when SVM classification is done only on cells in the external electric field (S7 Fig); in this case, the videos were randomly split into subgroups of  ( 1 , 1 , 1 )  for flat surfaces and  ( 1 , 1 , 2 )  for ridged surfaces, with 3 equivalent training/test splits constructed as before. Overall, these results show that the set of dictionary atoms shown in Fig 1 can be used to differentiate between cells moving on flat vs. ridged surfaces in the absence of the electric field, but not when the field is on. Apparently, the dependence of actin wave shapes on the nano-topography type diminishes or disappears when the external electric field is present.

Finally, we have checked whether SVM classification on dictionary atom-based feature vectors can be employed to distinguish cells in the presence vs. absence of the external electric field, regardless of the nano-topography type. In this case, the videos are randomly subdivided into groups of  ( 2 , 2 , 3 )  with the electric field and into groups of  ( 5 , 6 , 6 )  without the electric field; the rest of the procedure is as described above. The classification accuracy is also rather low in this case (S8 Fig), confirming our previous conclusion that the set of dictionary atoms in Fig 1 does not provide an adequate basis for classifying cellular images along the electric field axis.

### Application of scattering transforms to cellular images

For each video frame in the original videos, we compute zeroth- and first-order scattering coeﬃcients: $S_{0}(I),S_{1}^{j}(I)$, where *I* represents the set of pixel values in the original 2D image (see Materials and Methods for details). Altogether, there are n=1+LJ=49 filters, where *J* = 4 is the total number of scale parameters and L=12 is the total number of angular orientation parameters. Applying a zeroth- or first-order scattering transform to the original *M* × *M* image results in an output image of size $M∕2^{J}\;\times \;M∕2^{J}$. Since in our case M=512, the output images are 32×32 pixels. The set of wavelet filters in the scattering transform is designed to capture the presence of various features in the images. If the feature corresponding to a filter is present in the image, it is highlighted in the output image after applying that filter (see S9 and S10 Figs for representative examples).

**Fig 4:**
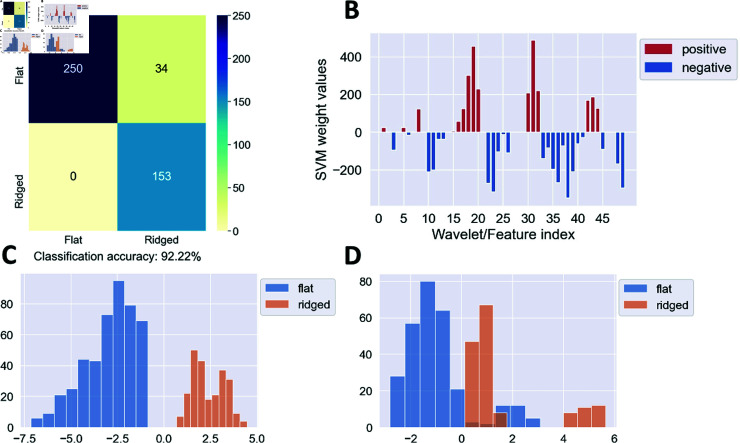
SVM classification of scattering transform feature vectors. The feature vectors are obtained from the images of *Dictyostelium* cells moving on flat vs. ridged surfaces in the absence of the external electric field. Shown are results for the training/test set split 1. Panel (A) is the confusion matrix for the SVM classification into the two nano-topography types (flat vs. ridged surfaces). Panel (B) shows SVM weights assigned to each scattering transform; the 1–49 numbering on the *x*-axis corresponds to the zeroth-order transform followed by the first-order transforms in the order displayed in S12 Fig. Note that positive/negative SVM weights describe features of cells on ridged/flat surfaces, respectively. Panel (C) is a histogram of SVM scores (Eq (1)) evaluated on all video frames from the training set; panel (D) is a histogram of SVM scores (Eq (1)) evaluated on all video frames from the test set.

In order to obtain feature vectors for image classification, we construct an *n*-dimensional vector *v* with components $v_{\alpha }=\sum_{i,j=1}^{32}X_{\alpha }(i,j)$ (α=1…n;n=49). Here, $X_{\alpha }(i,j)$ denotes the value of a pixel  ( *i* , *j* )  in the 32×32 output image obtained after applying wavelet filter *α* to the original image. After collecting feature vectors for all image frames in a given dataset, we carried out binary SVM classification as was done before for dictionary learning. Namely, we used the SVM classifier implemented in scikit-learn, with the *L*_1_ penalty on the fitting weights and *C* = 5000. First, we focused on classifying the videos by nano-topography type (flat or ridged) in the absence of the electric field. We used the same 3 training/test set splits as in the dictionary learning section. The results of the SVM classification on split 1 are shown in Fig 4A and on splits 2 and 3 in S11 Fig. We observe that the classification accuracy is somewhat lower than than obtained with dictionary learning. Note that all components of the feature vector *v* are non-negative due to the absolute magnitude operations in Eq (6). Thus, wavelet features corresponding to positive SVM weights are more prominent in the video frames from ridged surfaces, whereas wavelet features corresponding to negative SVM weights are more prominent in the video frames from flat surfaces (Fig 4B). The separation of SVM scores is excellent in the training set (Fig 4C) but less good in the test set (Fig 4D), leading to the misclassification of a subset of video frames on flat surfaces observed in Fig 4A. Examination of wavelets corresponding to top 3 positive and negative SVM weights (S12 Fig) shows that cells on ridged surfaces are characterized by horizontal or nearly horizontal features, whereas cells on flat surfaces are characterized by vertical or nearly vertical features. This is consistent with the dictionary learning analysis.

Similar to dictionary learning, we used classification of individual image frames to label entire videos by majority voting. For the split 1 test set, for videos of cells on flat surfaces, 3 out of 30 frames were misclassified in one video, all 31 frames were misclassified in another video, and no misclassification was observed in the remaining 2 videos. For videos of cells on ridged surfaces, 100% of the image frames were classified correctly. Thus, 5 out of 6 test set videos were classified correctly. For the split 2 test set, the only misclassification instance was in one video of cells on ridged surfaces, where 30 out of 120 frames were misclassified. Thus, all 5 videos are classified correctly by majority voting. Finally, in the split 3 test set significant misclassification was observed in both videos of cells on ridged surfaces (all 62 frames were misclassified in one video and 35 out of 60 frames were misclassified in the other video) and no misclassification was observed elsewhere, leading to 4 out of 6 videos classified correctly. This is again due to the relative lack of horizontal wave features in the two videos in question; it appears that scattering transforms are more sensitive to this than dictionary learning. We conclude that the classification accuracy of entire videos is somewhat lower with scattering transforms compared to dictionary learning.

As with dictionary learning, we have extended SVM classification into flat vs. ridged surfaces by including the frames from the videos with the electric field turned on into training and test sets. As expected, doing so has made the classification accuracy significantly worse (S13 Fig). The accuracy decreases again when only videos with the electric field present are considered (S14 Fig), showing that, similarly to dictionary learning, scattering transforms cannot be used to distinguish cells moving under the influence of the electric field by the nano-topography type. This conclusion is supported by the fact that classification of the cell videos by the presence or absence of the external electric field also shows relatively low accuracy (S15 Fig).

**Fig 5:**
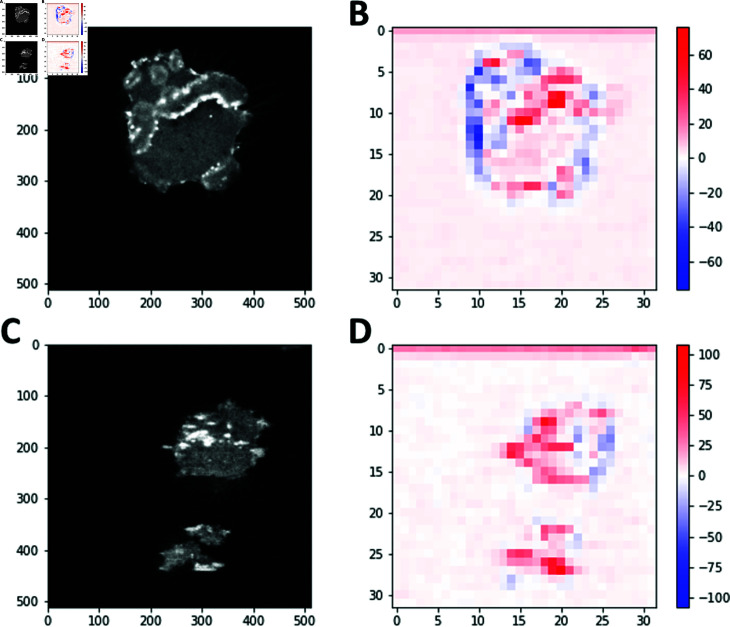
SVM-reconstructed cell images. The cells move on the flat (A,B) and ridged (C,D) surfaces, in the absence of the electric field. (A): A representative frame from a flat-surface video, (B): the corresponding SVM reconstruction $X^{r}(i,j)$. (C): A representative frame from a ridged-surface video, (D): the corresponding SVM reconstruction $X^{r}(i,j)$.

Next, we consider the product of the vector of weights and the feature vector in the SVM scoring function: $w\cdot v=\sum_{i,j}X^{r}(i,j)$, where $X^{r}(i,j)=\sum_{\alpha }w_{\alpha }X_{\alpha }(i,j)$ is the SVM-reconstructed image: the sum of *n* output 32×32 images from the scattering transform, weighted by their SVM weights. Two representative examples of $X^{r}(i,j)$ for cells moving on flat or ridged surfaces in the absence of the electric field are shown in Fig 5. Since $X_{\alpha }(i,j)\geq 0$, *∀* ⁡ *α*, the negative values of $X^{r}$, shown in blue in Fig 5B, 5D, identify pixel positions corresponding to flat-surface features. Conversely, the positive values of $X^{r}$, shown in red in Fig 5B, 5D, identify pixel positions corresponding to the features that are more prominent in ridged-surface frames. Note that the ridged-surface (red) features tend to be horizontal whereas the flat-surface (blue) features tend to be vertical in these examples, in line with the overall findings described above.

**Fig 6:**
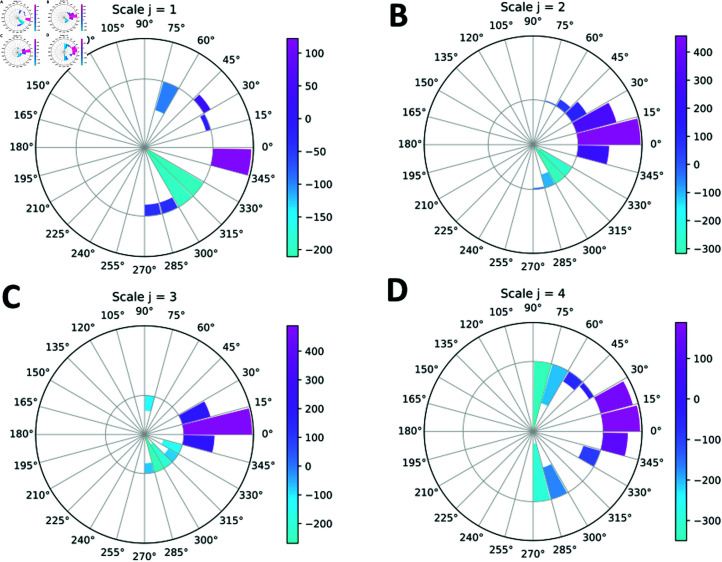
Angular plot of the SVM weight vector components for the Morlet wavelet filters. The filters are classified by different scales *j* = 1*…J* (*J* = 4) and angular orientation indices *ℓ* = 1*…L* (L=12). Each scale *j* corresponds to a separate panel (A)–(D). The angular orientation indices label angles between $90^{\circ }$ and $-90^{\circ }$ in $-15^{\circ }$ increments (cf. Materials and Methods). The inner and outer circles show negative and positive SVM weights, respectively.

Since the wavelet filters are labeled by the scale index *j* and the angular orientation index *ℓ* which labels angles in the $\left[90^{\circ },-90^{\circ }\right]$ range, it is convenient to display the SVM vector components on an angular plot (Fig 6). We observe that positive SVM weights, which are statistically more prominent in the frames from videos of cells on ridged surfaces, tend to correspond to angular orientations in the $\left[15^{\circ },-15^{\circ }\right]$ range and thus to horizontal and nearly horizontal features (S12 Fig). In contrast, negative SVM weights that characterize cells on flat surfaces correspond to more vertical features.

Finally, as was done earlier with dictionary learning, we checked the scattering transform performance on the enlarged dataset in which the original image frames were combined with the same images rotated counterclockwise by $90^{\circ }$, $180^{\circ }$, and $270^{\circ }$ (S16 Fig). In contrast to dictionary learning, we observe an appreciable loss of performance (cf. Fig 4A and S11 Fig on the one hand and S16A, S16D, and S16G Fig on the other). Apparently, the fixed set of features provided by the scattering transform is less suitable in this case than the adaptive set of dictionary learning features. The top features picked out by the SVM classifier are no longer vertical or horizontal—similarly to dictionary learning, the most discriminative features are oblique, likely to account for the mixture of horizontal and vertical nano-ridge orientations (S16 C, S16F, and S16I Fig).

### Classification using optical flow and tracking algorithm

**Fig 7:**
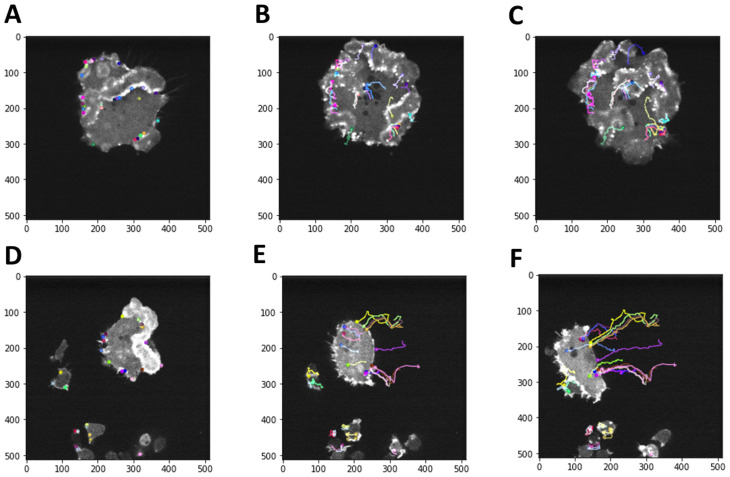
Mapping cell motion caused by the application of the external electric field: corner tracking and optical flow in videos of *Dictyostelium* cells. (A): The first frame from a video depicting cell motion on a flat surface with no external electric field. Colored circles indicate corners found using the Shi-Tomasi corner detection algorithm with the quality level *α* = 0 . 4 (Materials and Methods), resulting in 28 corner points detected. (B): The middle frame from the same video. (C): The last frame from the same video. Colored lines in (B) and (C) indicate optical flow trajectories for the set of corners shown in (A). (D): Same as (A), but for the first frame from a video depicting cell motion on a flat surface in the presence of the external electric field pointing to the left. Colored circles indicate corners found using the Shi-Tomasi corner detection algorithm with the quality level *α* = 0 . 4 (Materials and Methods), resulting in 28 corner points detected. (E): Same as (B), but for the middle frame from the video in (D). (F): Same as (C), but for the last frame from the video in (D). Colored lines in (E) and (F) indicate optical flow trajectories for the set of corners shown in (D). Each video frame is 107.52×107.52μm in size. Full videos of the cell motion and optical tracking are available as Movies S1 and S2.

We have implemented corner tracking using Lucas-Kanade optical flow algorithm (see Materials and Methods for details). We have found that tracking corners with optical flow is preferable to tracking all image pixels, allowing us to focus on a relatively sparse set of well-defined actin wave features. We observe that cells exhibit distinct patterns of motion with and without the external electric field (Fig 7; S1 and S2 Movies): there is no preferred direction of motion without the electric field (Fig 7A–7C), in contrast to the drift towards the left with the electric field (Fig 7D–7F). Therefore, we have focused first on the binary classification along the electric field axis, irrespective of the nano-topography type.

**Fig 8:**
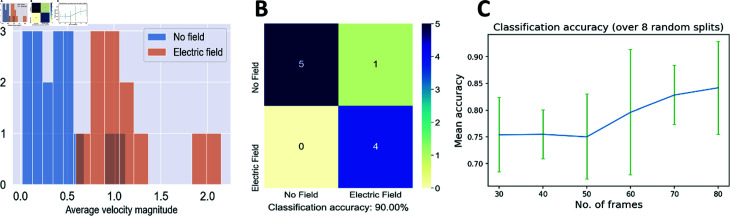
SVM classification of optical flow feature vectors. The feature vectors were obtained from the videos of *Dictyostelium* cells moving in the absence vs. presence of the external electric field, regardless of the nano-topography type. (A): A Histogram of average velocity magnitudes $\bar{v}$ for all the videos in our dataset. (B): Confusion matrix and classification accuracy for 10 videos in the test set (training/test set split 1). (C): Plot of the mean classification accuracy versus the number of sub-video frames *n*. The green vertical lines denote standard deviations.

Each video in our dataset yields a set of optical flow velocity vectors $\left(v_{x}v_{y}\right)$, one for each tracked corner and each pair of consecutive frames. In order to summarize these velocities in a low-dimensional representation, we compute ${\bar{v}}_{x}$ and ${\bar{v}}_{y}$ by averaging $v_{x}$ and $v_{y}$ over all the tracked points and all pairs of consecutive frames in a given video. We also compute $\bar{v}=\sqrt{{\bar{v}}_{x}^{2}+{\bar{v}}_{y}^{2}}$. We observe that even by itself, the average velocity magnitude $\bar{v}$ is an informative descriptor for separating videos with and without electric field (Fig 8A). Therefore, we construct a 3D feature vector $s=({\bar{v}}_{x}{\bar{v}}_{y}\bar{v})$ for each video and use a collection of these vectors in an SVM binary classifier. Our dataset includes both flat and ridged surfaces and in total consists of 17 videos in the absence of the electric field and 12 videos in the presence of the electric field (Table 1).

We divide all videos randomly into training and test sets in the 2 : 1 ratio (or the closest approximation to it); this procedure is repeated 3 times, resulting in 3 equivalent splits into test/training sets (labeled splits 1 , 2 , 3). As before, we use the SVM classifier implemented in scikit-learn, with the $L_{2}$ penalty on the fitting weights and C=10. We find that, as expected, classifying videos by the presence or absence of the external electric field using optical flow velocities as input yields reasonably accurate results, with 90% classification accuracy in split 1 (Fig 8B) and 80%, 100% classification accuracy in splits 2 and 3, respectively (S17 Fig). Interestingly, and consistent with Fig 8A, using $\bar{v}$ as a sole feature results in the SVM classification accuracy that is nearly as high: 90%, 90% and 80% for splits 1,2 and 3, respectively. This is noteworthy because, although the electric field is applied horizontally in all the videos, *v* is independent of the image rotation – it is the non-zero magnitudes of the velocities and not their orientations that enable the successful detection of the electric field.

Using entire videos for classification along the electric field axis results, for any given training/test split, in only 19 videos in the training set and 10 videos in the test set. We sought to increase the number of datapoints in both sets by subdividing each original video into smaller videos with a fixed number of frames. Specifically, we divided each original video into consecutive *n*-frame segments starting from the first frame; in the cases where the total number of frames was not exactly divisible by *n*, we also added the last *n* frames from each video to the dataset. For videos which have fewer than *n* frames, we added the entire video to the dataset. The resulting set of sub-videos was divided randomly into training and test sets in the 2 : 1 ratio (or the closest approximation to it); this procedure was repeated 8 times, resulting in 8 equivalent splits into test/training sets.

Using the same strategy as before, we computed a 3D feature vector *s* for each sub-video and carried out binary SVM classification, with the same settings as above, on the presence or absence of the external electric field. We find that the mean classification accuracy, averaged over the 8 splits, is high for longer videos (n=60,70,80) and becomes markedly lower when the videos are shorter (n=30,40,50) (Fig 8C). With n=80, the average classification accuracy is 76.8% when $\bar{v}$ is the sole classification feature, only a little worse than using the full 3D feature vector *s*. Thus, the magnitudes of the corner velocities are again the primary factor in detecting the motion of the cell under the influence of the external electric field. Overall, we conclude that optical flow-based feature vectors can be used to reliably detect the presence of electric fields from the patterns of actin wave motion in *Dictyostelium* cells. The detection can be based either on entire videos or on shorter sub-videos; there is no appreciable degradation of the performance, as long as the sub-videos contain sequences of at least 60 consecutive frames.

In contrast, the optical flow-based feature vectors cannot be used to predict the surface type (flat or ridged) when the videos both with and without external electric field are mixed together in the dataset. Using 3 random training/test splits in the approximately 2 : 1 ratio, we obtain a relatively low classification accuracy by the nano-topography type (S18 Fig). Thus, optical flow analysis is complementary to single-frame analysis employing either dictionary learning or scattering transforms.

## Discussion

In this work, we have used several state-of-the-art machine learning techniques originally developed in the field of computer vision to analyze video microscopy sequences of giant *D. discoideum* cells moving under four extracellular conditions: on flat or ridged nano-surfaces, and in the presence or absence of the external electric field. We have investigated two independent approaches which relied on analyzing *D. discoideum* videos frame by frame: dictionary learning [39–42] and scattering transforms [43–46]. While dictionary learning extracts a set of visual features (dictionary atoms) from the images themselves, scattering transforms employ a fixed set of wavelet transformations. In both cases, a sparse set of statistics is obtained for each image frame and then used to construct feature vectors for the SVM binary classifier. Our goals are two-fold: (i) develop a computational pipeline for the inference of cellular microenvironments (in this case, the nano-surface type and the presence of the electric field) solely on the basis of visual observations of moving cells; (ii) identify the corresponding set of actin wave visual features that are most prominent in differentiating between microenvironment types.

*A priori*, we expected scattering transforms to be more interpretable since they rely on a fixed set of features that are more easily visualized than data-dependent features extracted by dictionary learning (compare Fig 1 and S12 Fig). However, we find that both approaches are in qualitative agreement with one another: in the absence of the external electric field, video frames of cells moving on horizontally-oriented ridged surfaces contain more prominent horizontal features, while cells moving on flat surfaces are characterized by more vertical features. When the vertical and horizontal ridges are considered together, both dictionary learning and scattering transforms focus on more oblique features, with the latter exhibiting a loss of classification accuracy, probably due to the fact that the visual features are not adaptive in this case. These angled ridge features are similar to the nematic orientation order parameter (cf. Figs 1 and 6), and have to do with actin waveforms rather than actin fiber orientation. Taken together, these observations indicate that the topology of actin waves is directly affected by the nano-topography of the surface on which the cells move, in agreement with our previous observations [19–21].

Interestingly, these features can be used to classify cells by the nano-topography type only in the absence of the electric field (Figs 3 and 4). In this case, accurate frame-by-frame classification also yields accurate classification of the entire videos by majority voting. However, classification accuracy decreases significantly when both videos with and without electric fields are included into the dataset, or when the binary classification is carried out on the presence vs. absence of the electric field (S6 –S8, S13 –S15 Figs). Thus, single-frame analysis cannot be reliably used to study the biophysical effects of electric fields on cell motion, at least in *D. discoideum*. This observation is in agreement with our previous finding that electric fields profoundly modulate actin wave dynamics and physical properties. In the presence of the electric field, cells on ridged surfaces generate larger actin waves that resemble waves on flat surfaces [29].

In order to investigate the interplay between electric fields and actin wave dynamics, we turned to the optical flow technique, originally developed for analyzing video sequences of macroscopic objects in computer vision [49,50]. Optical flow analysis was previously used by some of the authors to extract statistical differences between electric field and no electric field conditions from movies of *D. discoideum* cells [29]. In that application, the microenvironmental conditions were known prior to the analysis. Here, we focus on the inverse problem: inference of the presence or absence of the electric field from the movies of cellular motion. In contrast with our previous work [21,29], here we first identify a key set of cellular features such as corners or edges and then track their motion in time using optical flow. The set of velocities extracted from the trajectories of the tracked features is used to carry out binary SVM classification on the environment type. We find that this approach yields reliable detection of the presence of the external electric field, regardless of whether the cells are moving on flat or ridged surfaces (Fig 8). In fact, just the knowledge of the velocity magnitudes, which are independent of the electric field direction, is suﬃcient for accurate classification. However, using velocity flow vectors of corner features is less effective in predicting the nano-topography type (S18 Fig). Thus, for the purposes of classifying the cellular microenvironment from visual observations the optical flow technique is complementary to the two single-frame techniques described above. We conclude that the presence of the external electric field can be most reliably detected by observing the movement of stable cellular features.

A standard alternative to the optical-flow based approach for detecting cell motion is provided by the particle image velocimetry (PIV) [58]. PIV gives a coarse-grained view of the dynamics and may not be well suited for the variety of features exhibited in fluorescence images of amorphous concentration fields, as demonstrated in a recent study [59]. In contrast, optical flow gives both useful pixel-scale information [21] and, as shown in this work, information on the motion of stable cellular features identified using corner detection methods.

In summary, we have carried out machine learning analysis of actin waves in electro-fused *D. discoideum* cells, focusing on the inference of the cellular microenvironment solely from images and videos provided by cellular microscopy. The microenvironments encompassed the type of nano-topography in contact with moving cells and the external stimulus provided by subjecting cells to the external electric field. We find that in the absence of the electric field, actin wave changes with the nano-topography type of the cell contact surfaces can be characterized using a set of static visual features. In contrast, turning the electric field on makes such static features less useful in classification – the presence of the electric field primarily manifests itself in actin wave dynamics, captured by analyzing video sequences rather than single frames. Overall, a combination of dictionary learning and optical flow appears to show the most promise in elucidating distinct external conditions that affect cellular motion. Our computational pipeline should be generally applicable to video microscopy imaging of cells in various biological and biomedical settings. We expect that our computer vision-based approach to discovering interpretable yet predictive visual features will enable quantitative descriptions of complex cellular phenotypes.

## Materials and methods

### Video microscopy dataset

All the data used in this work is available in our previous publication [29], which provides full experimental details of electrofusing giant cells, surface preparation, and electrotaxis. S19 Fig provides an overview of the experimental setup used to collect the video microscopy data. Briefly, the following procedures were employed:

Cell lines: Aggregation adenylyl cyclase null (ACA-) -LimE RFP cell lines, which do not have chemotaxis signal relay and express filamentous actin biosensors, were grown axenically in HL5 buffers. Before imaging, cells were washed twice with 17 mM Sorensen buffer (15 mM KH_2_PO_4_ and 2 mM Na_2_HPO_4_) to remove nutrients, and rolled in a 15 mL conical tube for 30 minutes before electrofusion. Afterwards, cells were seeded into an electrotaxis chamber for 2 hours before experiments.

**Fig 9:**
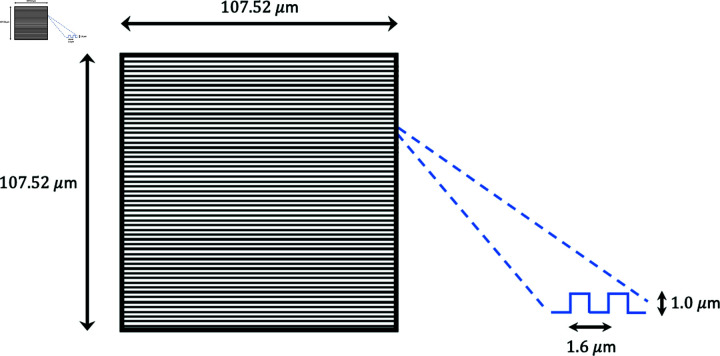
Schematic representation of the ridged nano-topography surface and its alignment with the image frame.

Surfaces: Both flat and ridged nano-topographies were fabricated using the same photopolymerizable resin. The ridged nano-topography surfaces were covered with 1.6 *μ*m-spaced nano-ridges at 1 *μ*m height; the ridges were aligned horizontally with respect to the image frame (Fig 9).

Electrotaxis: 20 V/cm DC electric field was applied to our customized 3D-printed electrotaxis chamber (dimension: 20 mm  ×  5 mm  ×  0.25 mm). The field always pointed horizontally in Fig 9, with several right-to-left reversals in the course of each experiment. Agar bridges were used to minimize toxic electrochemical products and maintain a stable pH environment.

Spinning disk microscopy: Time-lapse images of the RFP channel were recorded using a PerkinElmer spinning-disk microscope at a frame rate of 0.1 frames per second.

Video dataset: Our dataset consists of videos of giant electrofused *Dictyostelium* cells moving on flat or ridged surfaces, in the presence or absence of the external electric field. Each video frame in this study is 107.52×107.52μm in size. In the presence of the electric field, there are 3 videos of cells on flat surfaces, with 444, 460, and 126 frames; and 4 videos of cells on ridged surfaces with 675, 348, 699, and 656 frames. In the absence of the electric field, there are 12 videos of cells on flat surfaces and 5 videos of cells on ridged surfaces (the number of frames in each video is listed in Table 1). Altogether, we have 4551 video frames in our dataset. Each video frame is a grayscale image of the size M×M=512×512 pixels.

Note that the original videos with the electric field turned on contain several reversals of the electric field direction; after each change, there is a small time lag between the onset of the electric field and the onset of the cellular response to the electric field. Therefore, we have additionally subdivided each of these videos into sets of continuous frames where the cell motion has stabilized in response to the electric field with a fixed magnitude and direction. Each resulting sub-video has the number of frames ranging from 30 to 181; for videos without electric field, no subdivision was necessary and therefore the original video sequences were used (Table 1). The sub-videos listed in Table 1 were used for the optical flow analysis; the longer original videos were used for the dictionary learning and the scattering transform analysis.

### Dictionary learning

Dictionary learning (sparse coding) is a computational method which aims to find a sparse representation of data vectors in terms of a set of basis vectors that are learned from the data itself [39,41]. The idea is to reconstruct each data vector as faithfully as possible while employing very few active (i.e., non-zero) elements in the basis set.

Let us define a *d* × *N* data matrix *X*, where each of *N* columns of *X* represents a *d*-dimensional data vector $x_{i}$. Here, $d=N_{x}N_{y}$ for an image (or an image patch) of the size $N_{x}\;\times \;N_{y}$ pixels. The goal of dictionary learning is to factorize the data matrix *X* as follows:


$$\begin{array}{c} X=\left[\begin{array}{cccc} | & | & .... & |\\ x_{1} & x_{2} & .... & x_{N}\\ | & | & .... & | \end{array}\right]\approx DS=\left[\begin{array}{cccc} | & | & .... & |\\ D_{1} & D_{2} & .... & D_{M}\\ | & | & .... & | \end{array}\right]\left[\begin{array}{cccc} | & | & .... & |\\ s_{1} & s_{2} & .... & s_{N}\\ | & | & .... & | \end{array}\right], \end{array}$$ (2)

where *D* is a *d* × *M* dictionary matrix with each column $D_{i}$ representing one of *M* *d*-dimensional dictionary atoms, or basis vectors. *S* is an *M* × *N* matrix where each column $s_{i}$ contains the sparse code representation for the data vector $x_{i}$.

Dictionary learning is implemented by defining the following objective function:


$$\begin{array}{c} L=\frac{1}{2}||x_{i}-Ds_{i}|{|}_{2}^{2}+\lambda ||s_{i}|{|}_{1}, \end{array}$$ (3)

where the first term is the $L_{2}$ penalty for deviations between the data vectors and their dictionary learning representations (reconstruction error) and the second term is the $L_{1}$ penalty which induces sparseness in the code representation. In order to produce an optimal sparse representation, the objective function needs to be minimized with respect to the elements of both *D* and *S*:


min ⁡ D ∑i=1N min ⁡ siL, (4)

under the $D_{j}^{T}D_{j}=1$ constraint which prevents scaling transformations of the dictionary atoms. Such transformations could lower the $L_{1}$ penalty in Eq (3) through the trivial reduction of the overall magnitude of the matrix elements of *S* instead of inducing sparseness. Note that while *L* in Eq (4) is a convex function of either *D* or *S* separately, it is not a convex function of both variables together. Thus, to minimize *L* alternate optimization is performed by (i) keeping *D* fixed and minimizing *L* with respect to each $s_{i}$; (ii) keeping the latent representation *S* fixed and minimizing *L* with respect to *D* (see Supporting information (SI) Methods for details).

*Implementation.* We have implemented dictionary learning using the Dictionary Learning module in scikit-learn, with the minimization of *L* carried out by Block Coordinate Descent [60]. Note that the only parameter needed to be specified explicitly is the sparsity-inducing parameter *λ* in Eq (3).

### The scattering transform

The scattering transform is an image analysis technique which allows to extract a relatively compact and interpretable set of summary statistics starting from the raw image [44–46]. On the one hand, the scattering transform can be viewed as an extension of the power spectrum analysis, with expansions in terms of wavelets [43] rather than plane waves. On the other hand, it is conceptually related to convolutional neural networks (CNNs) which are routinely used in image analysis and classification [47,48,54,56]. However, unlike CNNs, the scattering transform yields more interpretable results and does not require training because wavelet convolutions do not have any fitting parameters. The scattering transform can be viewed as a non-linear transformation which decomposes a signal into a collection of translation-invariant coeﬃcients (summary statistics). It is widely used in signal processing and representation learning tasks in many areas of science [46].

The scattering transform has been recently reviewed and contrasted with other methods such as CNNs [46]. Briefly, at the second-order the transform yields a set of scattering coeﬃcients:


$$\begin{array}{c} S(I)=[S_{0}(I),S_{1}^{\lambda }(I),S_{2}^{\lambda_{1}\lambda_{2}}(I)], \end{array}$$ (5)

where *I* represents the original 2D image and $\lambda ,\lambda_{1},\lambda_{2}$ denote scale/orientation wavelet indices, as defined below.

The scattering coeﬃcients are given by


$$\begin{array}{cccc} & S_{0}(I)=I*\phi , & \\ & S_{1}^{\lambda }(I)=|I*\psi^{\lambda }|*\phi , & & \\ & S_{2}^{\lambda_{1}\lambda_{2}}(I)=||I*\psi^{\lambda_{1}}|*\psi^{\lambda_{2}}|*\phi . & & \end{array}$$ (6)

Here,  ∗  denotes the 2D convolution operation: $f*g=\int d^{2}uf(u)g(x-u)$ (*u* = ( *x* , *y* )  is the position in the image), *ϕ* is a low-pass filter such as the spatial average, and $\psi^{\lambda^{\prime }}$ is a wavelet kernel which acts as a band-pass filter and which is labeled with the scale/orientation index $\lambda^{\prime }$. Note that in practice, discrete convolutions are used and thus integrals are replaced with sums.

In Eq (6), the zeroth-order scattering coeﬃcient $S_{0}(I)$ is simply a convolution of the original image *I* with the low-pass filter. The first- and second-order scattering coeﬃcients are obtained by iteratively convolving *I* with a wavelet kernel, taking an absolute magnitude of the result and convolving with the low-pass filter at the end (extension to higher-order scattering coeﬃcients is straightforward). Taken together, the set of scattering coeﬃcients serves as a translation-invariant, compact representation of the image *I*. For 2D images, the wavelet index *λ* has two components: a scale index 1 ≤ *j* ≤ *J*, where the integer *J* > 0 specifies the overall averaging scale $2^{J}$ of the filter, and an angular orientation index 1 ≤ *ℓ* ≤ *L*, where *L* denotes the total number of angles used in the wavelet transform. For simplicity, we limit our analysis to the first-order transform.

*Implementation.* We used the wavelet scattering transform implemented in the Kymatio Python package (https://www.kymat.io) [61]. We employed 2D Morlet wavelets [43,45] with *J* = 4 and L=12 in the scattering transform (S12 Fig). Thus, there are 4 distinct spatial scales that determine the degree of localization of a given wavelet and 12 distinct angular orientations that correspond to $\left\{{\text{θ}}_{1},\theta_{2},\ldots ,\theta_{12}\}=\{90^{\circ },75^{\circ },\ldots ,-90^{\circ }\right\}$ (note that the scattering coeﬃcients are invariant with respect to a rotation through *π*).

The Morlet wavelets are defined as follows [45]. Let $u,u^{\prime }$ be two vectors in ${\mathbb{R}}^{2}$ describing  ( *x* , *y* )  positions in the image, and let $u\;\cdot \;u^{\prime }$ and  | *u* |  denote their inner product and the vector norm, respectively. The base Morlet wavelet *ψ* is given by


$$\begin{array}{c} \psi (u)=\alpha \;(e^{iu\cdot \xi }-\beta )\;e^{-|u{|}^{2}∕2\sigma^{2}}, \end{array}$$ (7)

where *α* is a normalization constant, *β* is adjusted so that  ∫ *ψ* ( *u* ) *du* = 0, and *ξ* and *σ* are constants fixed within the Kymatio package.

Let *G* be a discrete group of finite rotations in ${\mathbb{R}}^{2}$. Each group element *r* can be represented as a 2 × 2 matrix denoted by


 ( cos ⁡ θℓ− sin ⁡ θℓ sin ⁡ θℓ cos ⁡ θℓ),

where $\theta_{\ell }$ denotes distinct angular orientations (*ℓ* = 1*…L*).

Two-dimensional directional Morlet wavelets are obtained by rotating *ψ* in Eq (7) by *r* ∈ *G* and dilating it by $2^{j}$ for *j* ∈ *ℤ*:


$$\begin{array}{c} \psi^{\lambda }(u)=2^{-2j}\psi (2^{-j}r^{-1}u), \end{array}$$ (8)

where *λ* = ( *j* , *ℓ* )  is the composite wavelet index.

### Optical flow

*Lucas-Kanade algorithm.* Optical flow is a technique designed to track moving objects given a sequence of images (video frames) that are consecutive in time [49,50]. The idea is to estimate the velocity vectors for each pixel, or a group of pixels, using spatiotemporal gradients of pixel intensity. Let us denote the image intensity field at time *t* by *I* ( *x* , *y* , *t* ) , where *x* and *y* denote the 2D location of the pixel in the image. Assuming that the pixel’s brightness remains constant between *t* and *t* + *dt*, we have:


$$\begin{array}{c} I(x+dx,y+dy,t+dt)=I(x,y,t), \end{array}$$ (9)

where  ( *x* + *dx* , *y* + *dy* )  are the pixel coordinates at time *t* + *dt*. If *dt* is small and therefore  ( *dx* , *dy* )  are also small, we can use the first-order Taylor expansion to obtain the optical flow equation:


$$\begin{array}{c} \frac{\partial I}{\partial x}v_{x}+\frac{\partial I}{\partial y}v_{y}+\frac{\partial I}{\partial t}=0, \end{array}$$ (10)

where $v_{x}=\frac{dx}{dt}$ and $v_{y}=\frac{dy}{dt}$ are the *x* and *y* components of the optical flow velocity that is our quantity of interest. The optical flow velocity $\left(v_{x}v_{y}\right)$ cannot be directly estimated from Eq (10) because it is underconstrained. The Lucas-Kanade algorithm [50] is a standard approach to resolving this problem. It aims to estimate $\left(v_{x}v_{y}\right)$ by considering windows of fixed size (image patches) and assuming that the optical flow velocity is constant over that window:


$$\begin{array}{c} {\left(\frac{\partial I}{\partial x}\right)}_{i}v_{x}+{\left(\frac{\partial I}{\partial y}\right)}_{i}v_{y}+{\left(\frac{\partial I}{\partial t}\right)}_{i}=0 \end{array}$$ (11)

where *i* = 1*…N* enumerates all the pixels in the window and ${\left(\partial I∕\partial x\right)}_{i}$, ${\left(\partial I∕\partial y\right)}_{i}$, ${\left(\partial I∕\partial t\right)}_{i}$ are the spatial and temporal partial derivatives of the intensity $I_{i}$ for pixel *i*.

In order to estimate $v_{x},v_{y}$ for the window, the following objective function is minimized:


$$\begin{array}{c} \overset{N}{\underset{i=1}{\sum }}{\left[{\left(\frac{\partial I}{\partial x}\right)}_{i}v_{x}+{\left(\frac{\partial I}{\partial y}\right)}_{i}v_{y}+{\left(\frac{\partial I}{\partial t}\right)}_{i}\right]}^{2}, \end{array}$$ (12)

where the sum extends over all the pixels in the window. Repeated for all windows and all consecutive pairs of video frames, the Lucas-Kanade algorithm yields time-dependent optical flow vector field for the entire image (see SI Methods for details).

*Corner Detection.* Corners are regions in the image with an appreciable variation of intensity in all directions [51]. Computationally, corners can be found by applying a displacement vector $a^{T}=(a_{x}a_{y})$ to pixel intensities: $I(x,y)\to I(x+a_{x},y+a_{y})$. The weighted difference between the original and the displaced images is given by:


$$\begin{array}{c} E(a)=\underset{x,y}{\sum }w(x,y){\left[I(x+a_{x},y+a_{y})-I(x,y)\right]}^{2}, \end{array}$$ (13)

where the sum is over all the pixels in the image and *w* ( *x* , *y* )  is a rectangular or a Gaussian window function which assigns weights to the contribution of each pixel. If the displacement is small, Eq (13) can be simplified using the first-order Taylor expansion:


$$\begin{array}{c} E(a)≃a^{T}Ma, \end{array}$$ (14)

where


$$\begin{array}{c} M=\underset{x,y}{\sum }w(x,y)\left[\begin{array}{cc} \frac{\partial I}{\partial x}\frac{\partial I}{\partial x} & \frac{\partial I}{\partial x}\frac{\partial I}{\partial y}\\ \frac{\partial I}{\partial x}\frac{\partial I}{\partial y} & \frac{\partial I}{\partial y}\frac{\partial I}{\partial y} \end{array}\right] \end{array}$$ (15)

is a 2 × 2 matrix with the eigenvalues $\lambda_{1}$ and $\lambda_{2}$. Here, *∂I* ∕ *∂x* and *∂I* ∕ *∂y* denote the first derivatives of the pixel image intensity *I* ( *x* , *y* )  in the *x* and *y* directions, respectively. These derivatives are calculated using discrete approximations similar to the ones used in the optical flow calculations (see SI Methods for details). The magnitudes of the eigenvalues of *M* determine whether a region is a corner, an edge, or a flat area. Specifically, for corner regions one expects that the quantity *E*(*a*) is large for an arbitrary shift *a*, implying that both eigenvalues of *M* should be large.

In order to decide whether a window contains a corner or not, we use the Shi-Tomasi scoring function for corner detection [52,53]:


$$\begin{array}{c} R=\min(\lambda_{1},\lambda_{2}). \end{array}$$ (16)

If *R* is greater than a pre-specified threshold value, the region is considered to contain a corner. Other choices of the scoring function are available in the literature, such as that employed in the Harris corner detector [51].

*Implementation.* We use an optical optical flow-based feature tracking algorithm from OpenCV (https://docs.opencv.org/2.4.13.7/). Specifically, we employ the OpenCV function cv2.calcOpticalFlowPyrLK() to track features such as corner points in a video. To decide which features to focus on, we use the OpenCV function cv2.goodFeaturesToTrack(). This function uses the Shi-Tomasi corner-detection method described above [52,53] to find $N_{c}$ highest-scoring corner points in a video frame. For a grayscale image, we provide the number of corners $N_{c}$ we want to find, the quality level *α* (a number between 0 and 1 which denotes the minimum quality threshold below which all corners are rejected), and the minimum Euclidean distance $\ell_{min}$ between the detected corners. Here, we use $N_{c}=300$, *α* = 0 . 2, and $\ell_{min}=7$ pixels (except in Fig 7, where *α* = 0 . 4 was used to produce fewer corners in the interests of better visualization). To identify corners, we used a 10×10 pixel window in Eq (13). The function produces a list of all corners above the quality threshold, sorted by the quality score in the descending order. The resulting list is traversed from the top and for each corner all lower-quality corners closer than the Euclidean distance threshold are removed from the list. Finally, the function returns top $N_{c}$ entries from the list, or the entire list if it has $\leq N_{c}$ remaining entries.

We use the above procedure to find up to $N_{c}$ highest-scoring corners in the first video frame. These corners are tracked using Lucas-Kanade optical flow algorithm [50] implemented in cv2.calcOpticalFlowPyrLK(). This function requires the current frame, the corners detected in the current frame and the next frame as inputs, and produces the corner positions in the next frame accompanied by corner status indicators (1 if the corner is found in the next frame, and 0 otherwise). The procedure is then repeated iteratively over the entire video using a progressively smaller set of corners with corner status equal to 1; for corners with corner status equal to 0, the optical flow trajectory terminates. The entire procedure yields a set of optical flow trajectories with variable lengths. To increase the robustness of our predictions, the optical flow calculations are done using image pyramids – a successively downsampled collection of images derived from a single original image (https://docs.opencv.org/3.4/d4/d1f/tutorial_pyramids.html). The number of levels in the image pyramids is controlled via the parameter *maxLevel* in the function cv2.calcOpticalFlowPyrLK(), which we set equal to 3. The window size in the Lucas-Kanade algorithm is 30×30 pixels, centered on each corner point.

## Supporting information

Supporting methods Overview of the dictionary learning and the Lucas-Kanade algorithm for optical flow. (PDF)

Fig S1 Representative images of *Dictyostelium* cells in the absence of the external electric field. Panels (A–C): flat surfaces, panels (D–F): ridged surfaces with horizontally oriented ridges (see Materials and Methods for details). All image frames are 107 . 52 × 107 . 52*μm* in size. The smaller structures are single cells that failed to electrofuse into giant cells.
(PNG)

Fig S2 Representative images of *Dictyostelium* cells in the presence of the external electric field. Panels (A–C): flat surfaces, panels (D–F): ridged surfaces with horizontally oriented ridges (see Materials and Methods for details). All image frames are 107 . 52 × 107 . 52*μm* in size. The smaller structures are single cells that failed to electrofuse into giant cells.
(PNG)

Fig S3 SVM classification of feature vectors obtained by dictionary learning. The features characterize images of *Dictyostelium* cells on flat vs. ridged surfaces in the absence of the external electric field (training/test set split 2). Panel (A) is the confusion matrix for the SVM classification into two nano-topography types (flat/ridged surfaces). Panel (B) shows SVM weights assigned to each dictionary atom; the 1–30 numbering on the *x*-axis corresponds to the rows of atoms in Fig 1, starting from the upper left corner. Note that dictionary atoms characterized by positive/negative SVM weights describe features of cells on ridged/flat surfaces, respectively. Panel (C) is a histogram of SVM scores (Eq (1) in the main text) evaluated on all video frames from the training set; panel (D) is a histogram of SVM scores (Eq. (1) in the main text) evaluated on all video frames from the test set.
(PNG)

Fig S4 SVM classification of feature vectors obtained by dictionary learning. Same as Fig S3, but for the training/test set split 3.
(PNG)

Fig S5 SVM classification of feature vectors obtained by dictionary learning on the four-fold dataset with both original and rotated images. The features characterize images of *Dictyostelium* cells on flat vs. ridged surfaces in the absence of the external electric field. Panels A–C: training/test set split 1, panels D-F: split 2, panels G-I: split 3. Panels (A,D,G) are the confusion matrices for the SVM classification into two nano-topography types (all SVM parameters are as in the original dataset). Panels (B,E,H) show SVM weights assigned to each dictionary atom; the 1–30 numbering on the *x*-axis corresponds to the rows of atoms in panels (C,F,I) respectively, starting from the upper left corner (see Fig 1 for details). Note that the dictionary atoms characterized by positive/negative SVM weights describe features of cells on ridged/flat surfaces, respectively. In panels (C,F,I), the features corresponding to top 3 positive (red squares) and negative (blue squares) SVM weights are highlighted.
(PNG)

Fig S6 Confusion matrices for SVM classification on feature vectors obtained via dictionary learning. Shown are the results for training/test set splits 1–3 (panels A–C, respectively). The classification is for *Dictyostelium* cells moving on flat vs. ridged surfaces; the test and training sets are not conditioned on the presence or absence of the external electric field.
(PNG)

Fig S7 Confusion matrices for SVM classification on feature vectors obtained via dictionary learning. Shown are the results for training/test set splits 1–3 (panels A–C, respectively). The classification is for *Dictyostelium* cells moving on flat vs. ridged surfaces; the test and training sets are conditioned on the presence of the external electric field.
(PNG)

Fig S8 Confusion matrices for SVM classification on feature vectors obtained via dictionary learning. Shown are the results for training/test set splits 1–3 (panels A–C, respectively). The classification is for *Dictyostelium* cells moving in the presence vs. absence of the external electric field; the test and training sets are not conditioned on the nano-topography type.
(PNG)

Fig S9 Original image of a cell and its wavelet transforms. (A): An original 512 × 512 image frame from a video depicting cell motion on a flat surface in the absence of the electric field. (B,C,D): Output 32 × 32 images obtained after passing the image in panel (A) through 2D Morlet wavelet filters corresponding to *j* = 3, *ℓ* = 1; *j* = 3, *ℓ* = 6 and *j* = 3, *ℓ* = 12, respectively (see Fig S12 for a graphical depiction of Morlet wavelets). Bright spots in the transformed image correspond to the presence in the original image of the corresponding feature at that location.
(PNG)

Fig S10 Original image of a cell and its wavelet transforms. (A): An original 512 × 512 image frame from a video depicting cell motion on a ridged surface in the absence of the electric field. (B,C,D): Output 32 × 32 images obtained after passing the image in panel (A) through 2D Morlet wavelet filters corresponding to *j* = 3, *ℓ* = 1; *j* = 3, *ℓ* = 6 and *j* = 3, *ℓ* = 12, respectively (see Fig S12 for a graphical depiction of Morlet wavelets). Bright spots in the transformed image correspond to the presence in the original image of the corresponding feature at that location.
(PNG)

Fig S11 Confusion matrices for SVM classification on feature vectors obtained via scattering transforms. Shown are the results for training/test set splits 2 (A) and 3 (B). The classification is for *Dictyostelium* cells moving on flat vs. ridged surfaces in the absence of the external electric field.
(PNG)

Fig S12 A set of 2D Morlet wavelet filters. Each panel shows a filter characterized by a scale index 1 ≤ *j* ≤ *J* and an angle index 1 ≤ *ℓ* ≤ *L*, with *J* = 4 and *L* = 12. Color saturation and hue denote the magnitude and the phase of each filter, respectively (see the Kymatio package: https://www.kymat.io for details). Highlighted are the features corresponding to top 3 positive (red squares) and negative (blue squares) SVM weights in Fig 4.
(PNG)

Fig S13 Confusion matrices for SVM classification on feature vectors obtained via scattering transforms. Shown are the results for training/test set splits 1–3 (panels A–C, respectively). The classification is for *Dictyostelium* cells moving on flat vs. ridged surfaces; the test and training sets are not conditioned on the presence or absence of the external electric field.
(PNG)

Fig S14 Confusion matrices for SVM classification on feature vectors obtained via scattering transforms. Shown are the results for training/test set splits 1–3 (panels A–C, respectively). The classification is for *Dictyostelium* cells moving on flat vs. ridged surfaces; the test and training sets are conditioned on the presence of the external electric field.
(PNG)

Fig S15 Confusion matrices for SVM classification on feature vectors obtained via scattering transforms. Shown are the results for training/test set splits 1–3 (panels A–C, respectively). The classification is for *Dictyostelium* cells moving in the presence vs. absence of the external electric field; the test and training sets are not conditioned on the nano-topography type.
(PNG)

Fig S16 SVM classification of feature vectors obtained by scattering transforms applied to the four-fold dataset with both original and rotated images. The features characterize images of *Dictyostelium* cells on flat vs. ridged surfaces in the absence of the external electric field. Panels A–C: training/test set split 1, panels D–F: split 2, panels G–I: split 3. Panels (A,D,G) are the confusion matrices for the SVM classification into two nano-topography types (all SVM parameters are as in the original dataset). Panels (B,E,H) show SVM weights assigned to each scattering transform; the 1–49 numbering on the *x*-axis corresponds to the zeroth-order transform followed by the first-order transforms in the order displayed in S12 Fig. Note that the positive/negative SVM weights describe features of cells on ridged/flat surfaces, respectively. In panels (C,F,I), the features corresponding to top 3 positive (red squares) and negative (blue squares) SVM weights are highlighted.
(PNG)

Fig S17 Confusion matrices for SVM classification on feature vectors obtained via optical flow analysis. Shown are the results for random training/test set splits 2 (A) and 3 (B). The classification is for *Dictyostelium* cells moving in the presence vs. absence of the external electric field, regardless of the nano-topography type.
(PNG)

Fig S18 Confusion matrices for SVM classification on feature vectors obtained via optical flow analysis. Shown are the results for random training/test set splits 1–3 (panels A–C, respectively). The classification is for *Dictyostelium* cells moving on flat vs. ridged surfaces; the test and training sets are not conditioned on the presence or absence of the external electric field.
(PNG)

Fig S19 Experimental setup. There are four steps in the experimental procedure. (i): Isolate *D. discoideum* cells from the cell culture. The cell media is centrifuged twice to remove the nutrients from the culture media. (ii): Cells are set in the 15 mL centrifuge tube and the tube is rolled for 30 min to cluster single cells together. (iii): After rolling, three electric pulses at the voltage of 1 kV are applied to the cells to open up the cell membranes and form giant cells by fusion. (iv): After electrofusion, the cells are transferred to the nano-ridges and incubated for 1–2 h before imaging. Afterwards, the chamber is transferred to the imaging core and the data are collected using a spinning disk confocal microscope.
(PNG)

Movie S1 Video of the cell motion, corner tracking, and optical flow in the absence of the external electric field. Fig 7A–7C shows the first, middle, and last frames from this video.
(TIFF)

Movie S2 Video of the cell motion, corner tracking, and optical flow in the presence of the external electric field. Fig 7D–7F shows the first, middle, and last frames from this video.
(TIFF)
