# Supplementary material for: Spatiotemporal feature learning for actin dynamics
Source: PLoS One. 2025 Mar 5;20(3):e0318036. doi: 10.1371/journal.pone.0318036 (PMC11882080; doi:10.1371/journal.pone.0318036)
Supplement: Supporting methods — (PDF) [file pone.0318036.s022.pdf]

Supporting information Methods: Spatiotemporal feature learning for actin dynamics

Introduction to Dictionary Learning

Objective function minimization. In dictionary learning, the goal is to minimize the following objective function:

$$\mathcal{L} = \frac{1}{2}||x_i - Ds_i||_2^2 + \lambda||s_i||_1, \tag{1}$$

where the first term is the L_2 penalty for deviations between the d-dimensional data vectors x_i and their dictionary learning representations Ds_i (D is a matrix where each column is a dictionary basis vector and s_i contains the sparse code representation for the data vector x_i). The second term is the L_1 penalty which induces sparseness in the code representation. In order to produce an optimal sparse representation, the objective function in Eq. (1) needs to be minimized with respect to the elements of D as well as s_i. This is done by alternate minimization of $\mathcal{L}$ with respect to (I) s_i (while keeping D fixed) and (II) D (while keeping s_i fixed). Note that each round of alternate minimization is a convex problem and therefore can be implemented using gradient-based minimizers.

I. Keeping D fixed, $\mathcal{L}$ is minimized with respect to each s_i:

$$\tilde{s}_i = \arg\min_{s_i} \mathcal{L}. \tag{2}$$

The gradient of $\mathcal{L}$ is given by

$$\nabla_{s_i}\mathcal{L} = D^T(Ds_i - x_i) + \lambda \text{sign}(s_i). \tag{3}$$

For a single component of each sparse code s_i^k ($k = 1 \dots M$), Eq. (3) becomes

$$\frac{\partial \mathcal{L}}{\partial s_i^k} = \sum_{m,n} D_{mk}(D_{mn}s_i^n - x_i^m) + \lambda \text{sign}(s_i^k). \tag{4}$$

The gradient in Eq. (4) is discontinuous at $s_i^k = 0$ due to the second term on the right-hand side. Since one expects most of the components of s_i to be zero due to sparsity, the following algorithm known as the ISTA (Iterative Shrinkage and Thresholding Algorithm) is used [1]:

$s_i^k \leftarrow s_i^k - \alpha \sum_{m,n} D_{km}(D_{mn}s_i^n - x_i^m)$
if $\text{sign}(s_i^k) \neq \text{sign}(s_i^k - \alpha\lambda \text{sign}(s_i^k))$ **then**
    $s_i^k = 0$
else
    $s_i^k \leftarrow s_i^k - \alpha\lambda \text{sign}(s_i^k)$
end if

These iterations are repeated until convergence. Here, α determines the gradient descent step size.

II . Given the latent representation S, $\mathcal{L}$ is minimized with respect to D:

$$\tilde{D} = \arg\min_{D} \mathcal{L}, \tag{5}$$

subject to the unit norm constraint $D_j^T D_j = 1$ $(j = 1 \dots M)$.

For optimization, one can use the Projected Gradient Descent algorithm (`https://tlienart.github.io/posts/2018/10/10-projected-gradient-descent/`):

$$D \leftarrow D - \alpha' \sum_{i=1}^{N} (Ds_i - x_i) s_i^T. \tag{6}$$

Here, α' determines the gradient descent step size. Normalization is explicitly enforced at each step:

$$D_j \leftarrow \frac{D_j}{||D_j||_2}. \tag{7}$$

The iterations are repeated until convergence.

By alternating gradient minimization in steps I and II, one can learn both the dictionary D and the set of sparse codes S adapted to the data set under consideration. Other minimization algorithms can be used for dictionary learning, including Block Coordinate Descent which does not require a gradient descent step size [2], and the Dual Lagrangian formalism [3].

Probabilistic interpretation. Dictionary learning can be viewed as a linear generative model [4]:

$$x = Ds + n, \tag{8}$$

where x is a data vector, s is its sparse representation, and n is the noise term. Assuming that n is Gaussian-distributed with zero mean and the covariance matrix $\sigma^2 I$, we obtain the likelihood of observing x given s:

$$P(x|s) \propto e^{-\frac{||x - Ds||_2^2}{2\sigma^2}}. \tag{9}$$

Introducing a sparsity-inducing prior,

$$P(s) \propto \prod_k e^{-\lambda |s^k|}, \tag{10}$$

where $k = 1 \dots M$ denotes a component of vector s, we obtain the following posterior distribution for s:

$$P(s|x) \propto e^{-\frac{||x - Ds||_2^2}{2\sigma^2} - \sum_k \lambda |s^k|}. \tag{11}$$

Maximizing the posterior in Eq. (11) with respect to D and s is equivalent to minimizing the objective function $\mathcal{L}$ in Eq. (1).

Lucas-Kanade algorithm for optical flow

In Lucas-Kanada algortihm, the goal is to find (v_x, v_y) (the components of the velocity vector for a small window of pixels) by minimizing the following objective function:

$$\sum_{i=1}^{N} \left[\left(\frac{\partial I}{\partial x}\right)_i v_x + \left(\frac{\partial I}{\partial y}\right)_i v_y + \left(\frac{\partial I}{\partial t}\right)_i \right]^2, \tag{12}$$

where the sum extends over all pixels in the window and $(\partial I/\partial x)_i$, $(\partial I/\partial y)_i$, $(\partial I/\partial t)_i$ are the spatial and temporal partial derivatives of the intensity I_i for pixel i.

The objective function in Eq. (12) is quadratic in v_x and v_y and therefore its minimization can be carried out explicitly [5]. Let us define

$$A = \begin{bmatrix} | & | \\ (\frac{\partial I}{\partial x})_i & \left(\frac{\partial I}{\partial y}\right)_i \\ | & | \end{bmatrix}, \; b = -\begin{bmatrix} | \\ (\frac{\partial I}{\partial t})_i \\ | \end{bmatrix}. \tag{13}$$

Then the values of v_x and v_y that minimize the objective function in Eq. (12) are given by:

$$\begin{bmatrix} v_x \\ v_y \end{bmatrix} = (A^T A)^{-1} A^T b = \begin{bmatrix} \sum_{i=1}^{N} (\frac{\partial I}{\partial x})_i^2 & \sum_{i=1}^{N} (\frac{\partial I}{\partial y})_i (\frac{\partial I}{\partial x})_i \\ \sum_{i=1}^{N} (\frac{\partial I}{\partial x})_i (\frac{\partial I}{\partial y})_i & \sum_{i=1}^{N} (\frac{\partial I}{\partial y})_i^2 \end{bmatrix}^{-1} \begin{bmatrix} -\sum_{i=1}^{N} (\frac{\partial I}{\partial x})_i (\frac{\partial I}{\partial t})_i \\ -\sum_{i=1}^{N} (\frac{\partial I}{\partial y})_i (\frac{\partial I}{\partial t_i})_i \end{bmatrix} \tag{14}$$

It is important to note that, depending on the pixel pattern in the window, the matrix product $A^T A$ may not have a numerically stable inverse, e.g. when one has edge-like structures. Thus, Eq. (14) is typically regularized:

$$\begin{bmatrix} v_x \\ v_y \end{bmatrix} = (A^T A + \lambda I)^{-1} A^T b, \tag{15}$$

where λ is the parameter that controls the strength of L_2 regularization in the cost function. In other words, Eq. (15) is equivalent to adding the $\lambda(v_x^2 + v_y^2)$ regularizer term to Eq. (12).

To calculate the spatial and time derivatives of the intensity field, discrete approximations are employed. For an image frame represented as a 2D array, the spatial gradients can be computed using second-order central differences for the interior points and either first- or second-order one-sided differences (forward or backward) at the boundaries. Other methods employ derivative masks of fixed size such as convolution kernels of size 2×2 or 3×3, so that the spatial derivatives at every pixel location are obtained by a 2D convolution between these kernels and the image. For the time derivative, we consider two consecutive image frames I_1 and I_2 and perform a convolution with a 2D kernel given by $\frac{1}{4}\begin{bmatrix} 1 & 1 \\ 1 & 1 \end{bmatrix}$. We pad the images with zeros at the boundaries so that the convolved outputs have the same size as the images. Denoting the convolved outputs as $\tilde{I_1}$ and $\tilde{I_2}$, the pixel-by-pixel 2D signal for the time derivative is given by $\tilde{I_2} - \tilde{I_1}$.

The Lucas-Kanade algorithm generally produces a dense optical flow by creating fixed-size windows around every pixel. One can also evaluate optical flow vectors for a sparse set of features such as corners or edges, tracking those features over time frames.

References

1. Beck A, Teboulle M. A Fast Iterative Shrinkage-Thresholding Algorithm for Linear Inverse Problems. SIAM J Img Sci. 2009;2:183–202.
2. Blondel M, Seki K, Uehara K. Block Coordinate Descent Algorithms for Large-Scale Sparse Multiclass Classification. Machine Learning. 2013;93:31–52.
3. Lee H, Battle A, Raina R, Ng AY. Efficient Sparse Coding Algorithms. NIPS'06. Cambridge, MA, USA: MIT Press; 2006. p. 801–808.
4. Olshausen BA, Field DJ. Sparse coding with an overcomplete basis set: A strategy employed by V1? Vision Research. 1997;37:3311–3325.
5. Bishop CM. Pattern Recognition and Machine Learning. New York, NY: Springer; 2006.
