# Supplementary figures and images for: Spatiotemporal feature learning for actin dynamics
Source: PLoS One. 2025 Mar 5;20(3):e0318036. doi: 10.1371/journal.pone.0318036 (PMC11882080; doi:10.1371/journal.pone.0318036)

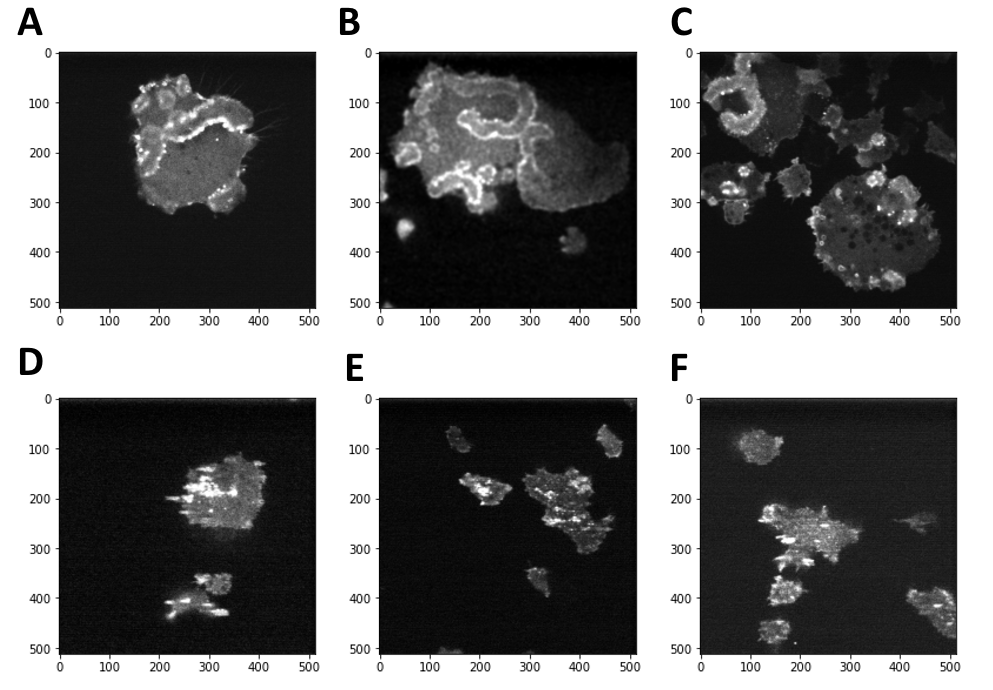

Supplement: Fig S1 — Panels (A–C): flat surfaces, panels (D–F): ridged surfaces with horizontally oriented ridges (see Materials and Methods for details). All image frames are 107 . 52 × 107 . 52μm in size. The smaller structures are single cells that failed to electrofuse into giant cells. (PNG) [file pone.0318036.s001.png]

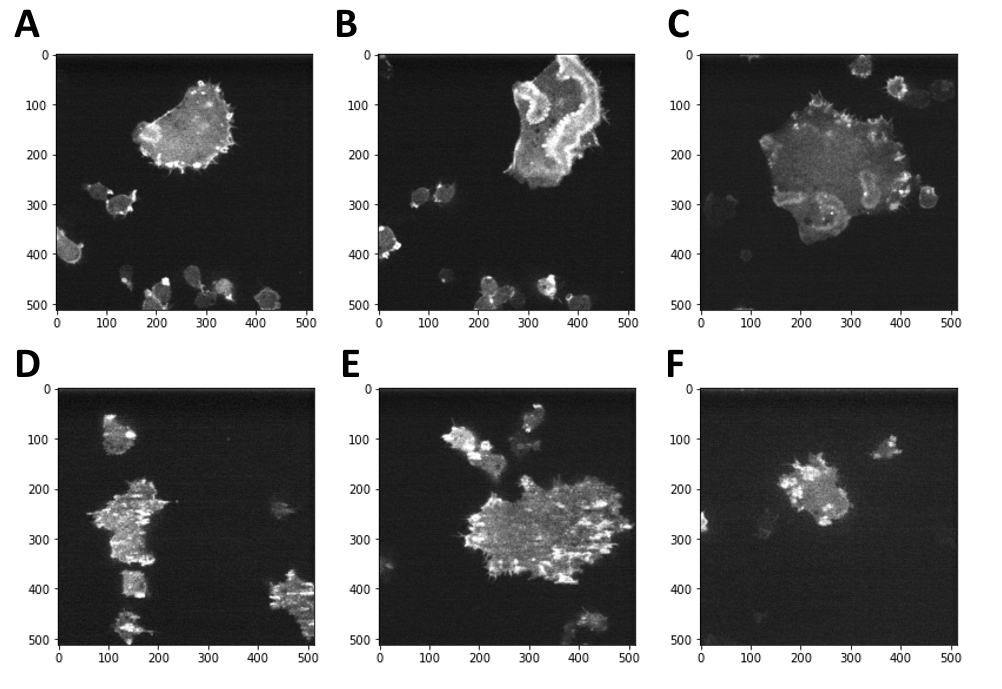

Supplement: Fig S2 — Panels (A–C): flat surfaces, panels (D–F): ridged surfaces with horizontally oriented ridges (see Materials and Methods for details). All image frames are 107 . 52 × 107 . 52μm in size. The smaller structures are single cells that failed to electrofuse into giant cells. (PNG) [file pone.0318036.s002.png]

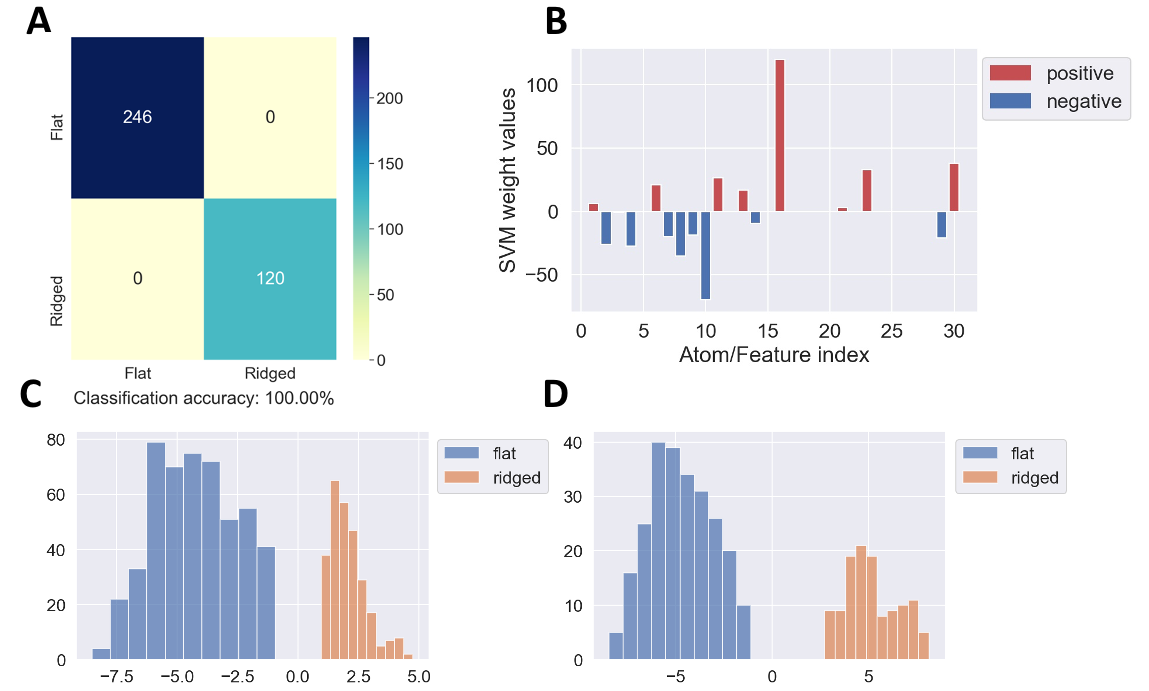

Supplement: Fig S3 — The features characterize images of Dictyostelium cells on flat vs. ridged surfaces in the absence of the external electric field (training/test set split 2). Panel (A) is the confusion matrix for the SVM classification into two nano-topography types (flat/ridged surfaces). Panel (B) shows SVM weights assigned to each dictionary atom; the 1–30 numbering on the x-axis corresponds to the rows of atoms in Fig 1, starting from the upper left corner. Note that dictionary atoms characterized by positive/negative SVM weights describe features of cells on ridged/flat surfaces, respectively. Panel (C) is a histogram of SVM scores (Eq (1) in the main text) evaluated on all video frames from the training set; panel (D) is a histogram of SVM scores (Eq. (1) in the main text) evaluated on all video frames from the test set. (PNG) [file pone.0318036.s003.png]

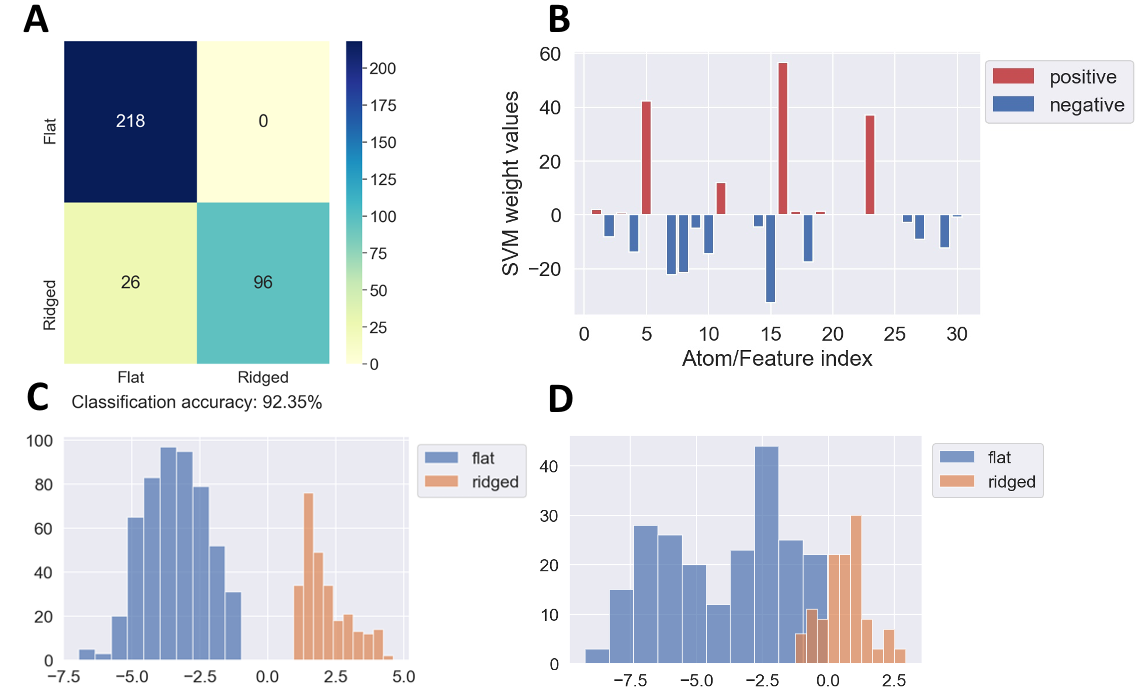

Supplement: Fig S4 — Same as Fig S3, but for the training/test set split 3. (PNG) [file pone.0318036.s004.png]

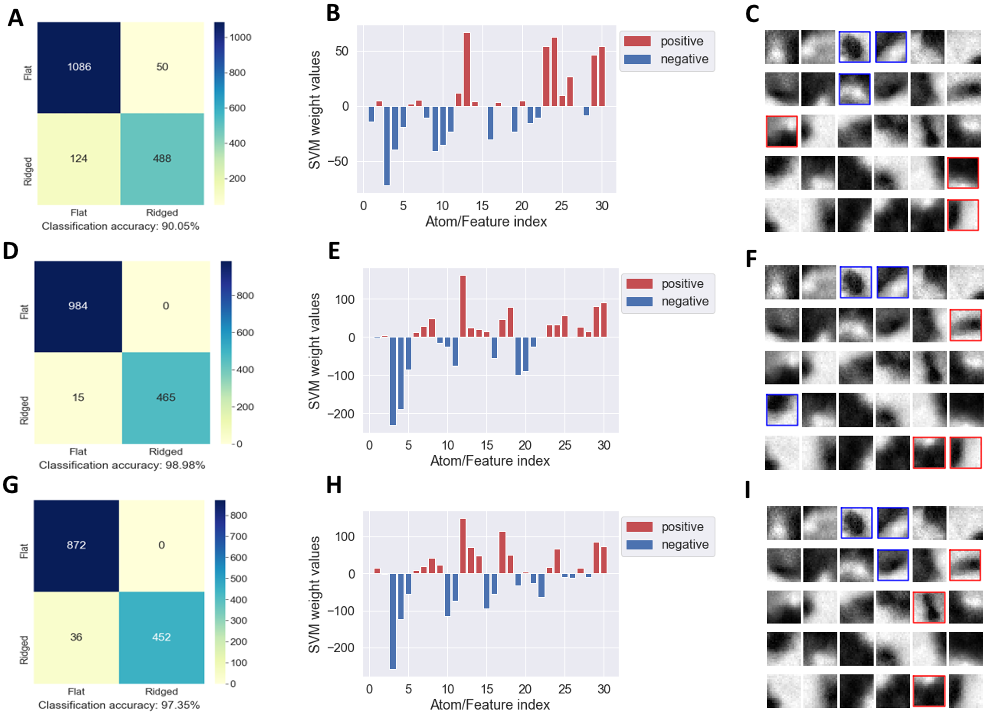

Supplement: Fig S5 — both original and rotated images. The features characterize images of Dictyostelium cells on flat vs. ridged surfaces in the absence of the external electric field. Panels A–C: training/test set split 1, panels D-F: split 2, panels G-I: split 3. Panels (A,D,G) are the confusion matrices for the SVM classification into two nano-topography types (all SVM parameters are as in the original dataset). Panels (B,E,H) show SVM weights assigned to each dictionary atom; the 1–30 numbering on the x-axis corresponds to the rows of atoms in panels (C,F,I) respectively, starting from the upper left corner (see Fig 1 for details). Note that the dictionary atoms characterized by positive/negative SVM weights describe features of cells on ridged/flat surfaces, respectively. In panels (C,F,I), the features corresponding to top 3 positive (red squares) and negative (blue squares) SVM weights are highlighted. (PNG) [file pone.0318036.s005.png]

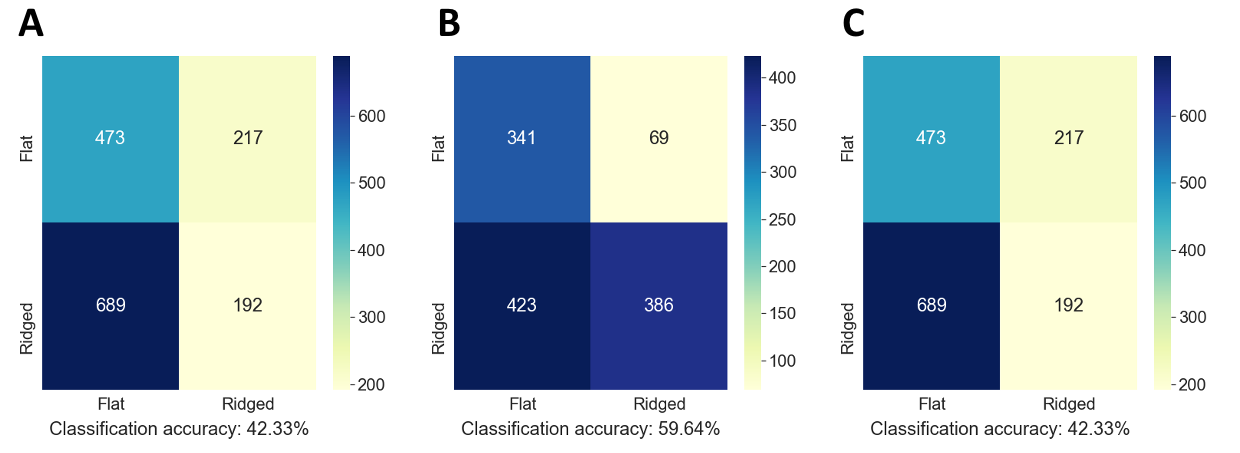

Supplement: Fig S6 — Shown are the results for training/test set splits 1–3 (panels A–C, respectively). The classification is for Dictyostelium cells moving on flat vs. ridged surfaces; the test and training sets are not conditioned on the presence or absence of the external electric field. (PNG) [file pone.0318036.s006.png]

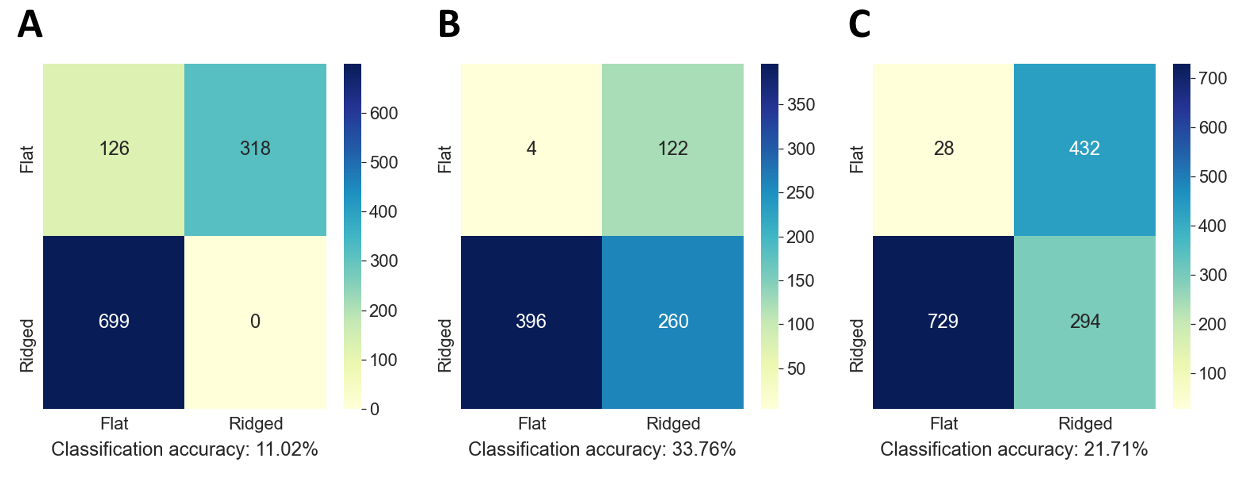

Supplement: Fig S7 — Shown are the results for training/test set splits 1–3 (panels A–C, respectively). The classification is for Dictyostelium cells moving on flat vs. ridged surfaces; the test and training sets are conditioned on the presence of the external electric field. (PNG) [file pone.0318036.s007.png]

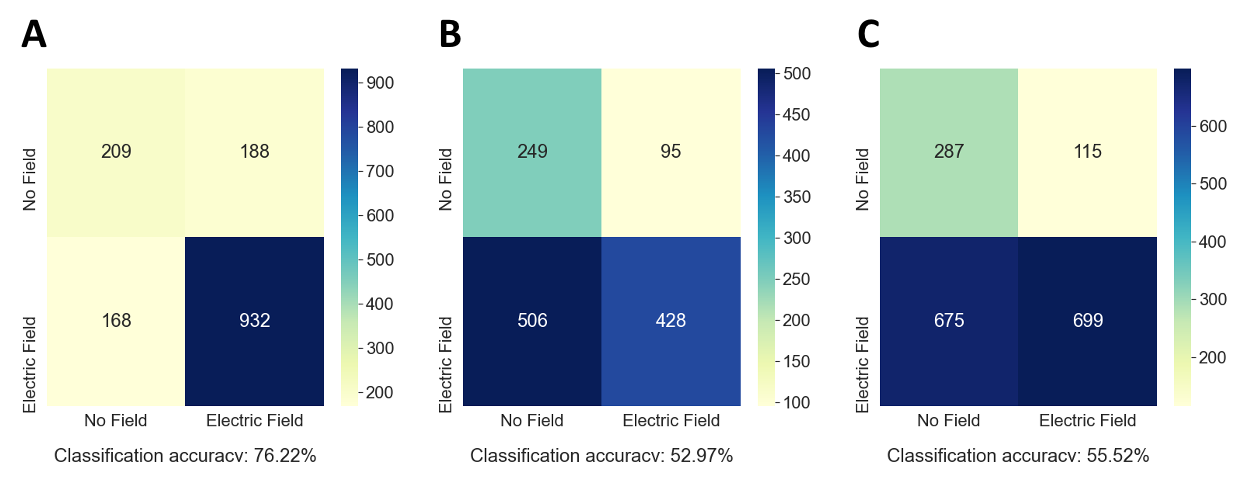

Supplement: Fig S8 — Shown are the results for training/test set splits 1–3 (panels A–C, respectively). The classification is for Dictyostelium cells moving in the presence vs. absence of the external electric field; the test and training sets are not conditioned on the nano-topography type. (PNG) [file pone.0318036.s008.png]

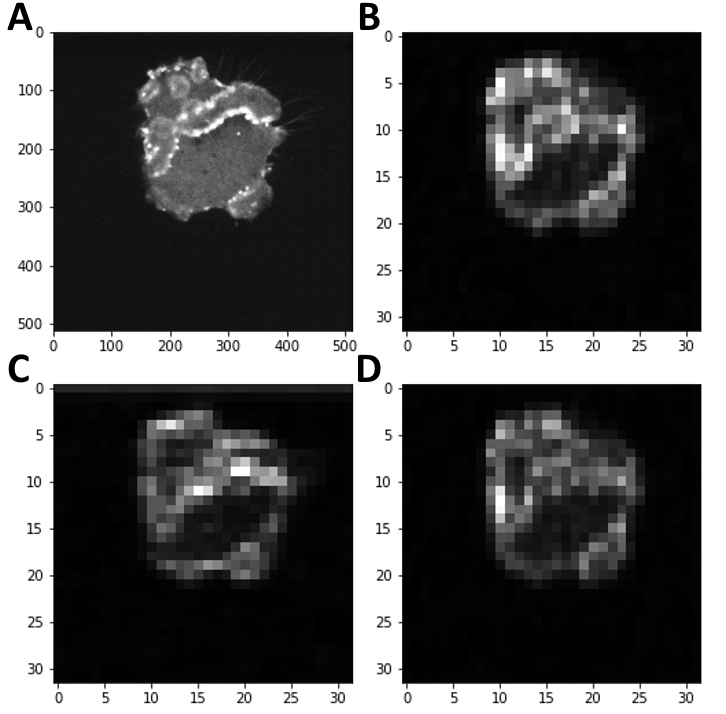

Supplement: Fig S9 — (A): An original 512 × 512 image frame from a video depicting cell motion on a flat surface in the absence of the electric field. (B,C,D): Output 32 × 32 images obtained after passing the image in panel (A) through 2D Morlet wavelet filters corresponding to j = 3, ℓ = 1; j = 3, ℓ = 6 and j = 3, ℓ = 12, respectively (see Fig S12 for a graphical depiction of Morlet wavelets). Bright spots in the transformed image correspond to the presence in the original image of the corresponding feature at that location. (PNG) [file pone.0318036.s009.png]

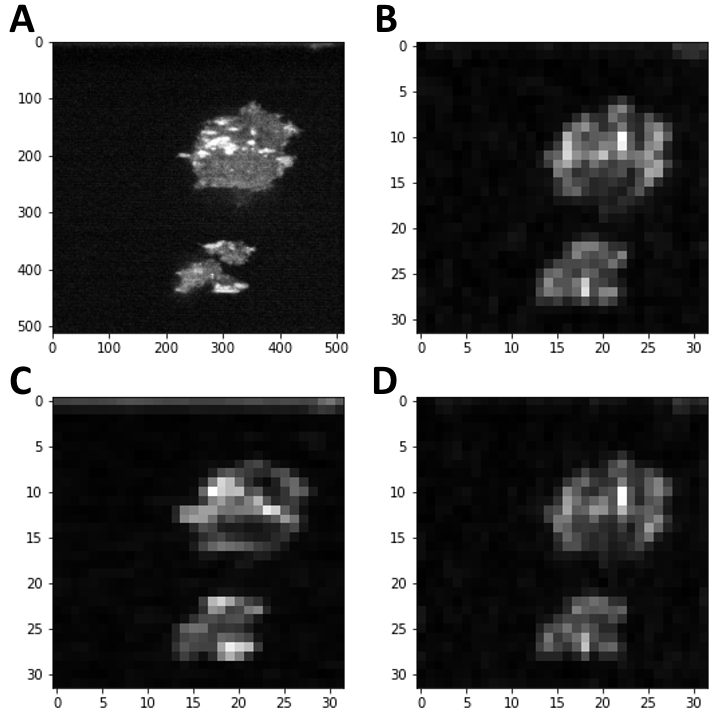

Supplement: Fig S10 — (A): An original 512 × 512 image frame from a video depicting cell motion on a ridged surface in the absence of the electric field. (B,C,D): Output 32 × 32 images obtained after passing the image in panel (A) through 2D Morlet wavelet filters corresponding to j = 3, ℓ = 1; j = 3, ℓ = 6 and j = 3, ℓ = 12, respectively (see Fig S12 for a graphical depiction of Morlet wavelets). Bright spots in the transformed image correspond to the presence in the original image of the corresponding feature at that location. (PNG) [file pone.0318036.s010.png]

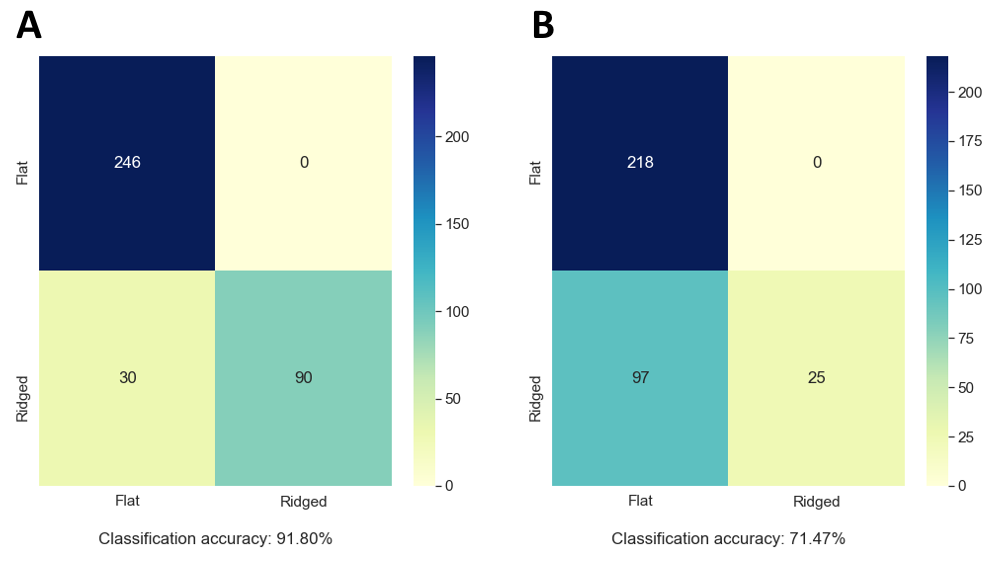

Supplement: Fig S11 — Shown are the results for training/test set splits 2 (A) and 3 (B). The classification is for Dictyostelium cells moving on flat vs. ridged surfaces in the absence of the external electric field. (PNG) [file pone.0318036.s011.png]

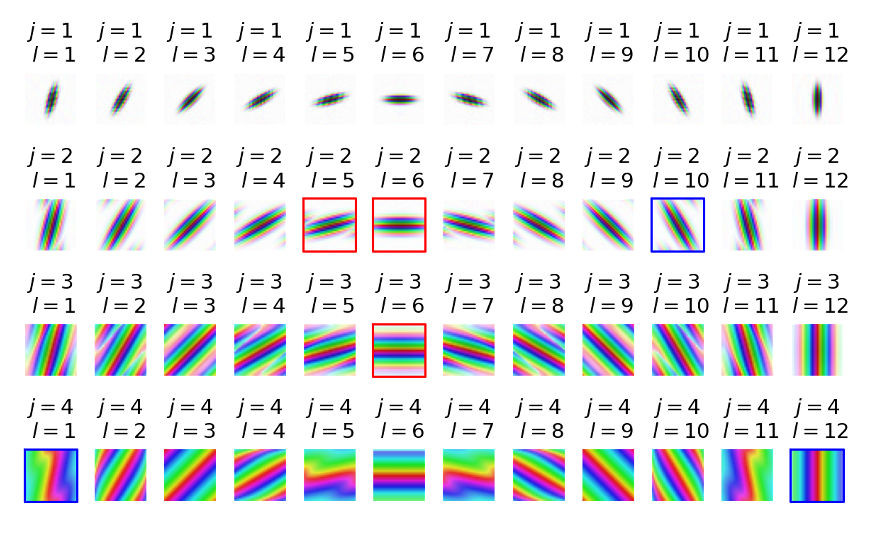

Supplement: Fig S12 — Each panel shows a filter characterized by a scale index 1 ≤ j ≤ J and an angle index 1 ≤ ℓ ≤ L, with J = 4 and L = 12. Color saturation and hue denote the magnitude and the phase of each filter, respectively (see the Kymatio package: https://www.kymat.io for details). Highlighted are the features corresponding to top 3 positive (red squares) and negative (blue squares) SVM weights in Fig 4. (PNG) [file pone.0318036.s012.png]

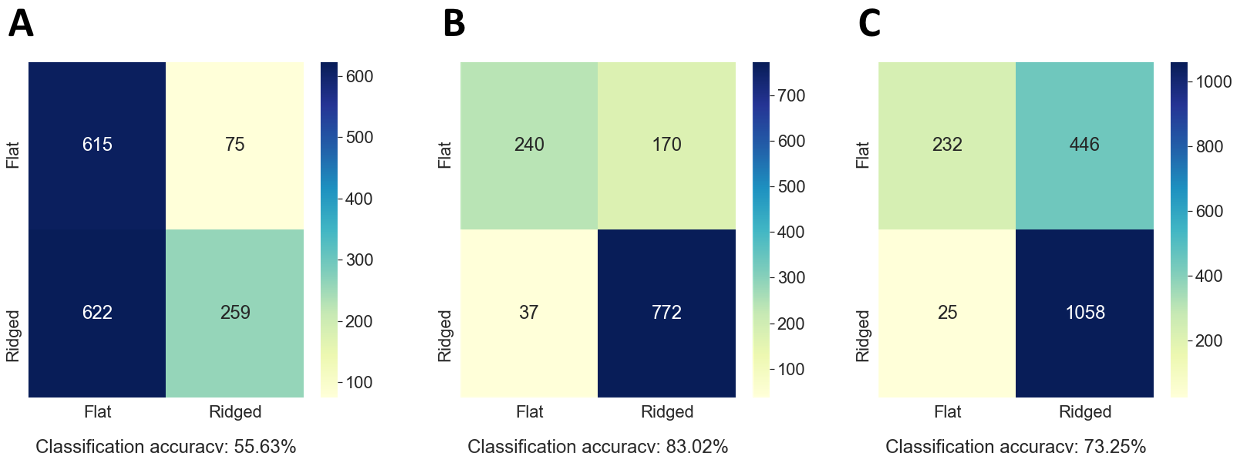

Supplement: Fig S13 — Shown are the results for training/test set splits 1–3 (panels A–C, respectively). The classification is for Dictyostelium cells moving on flat vs. ridged surfaces; the test and training sets are not conditioned on the presence or absence of the external electric field. (PNG) [file pone.0318036.s013.png]

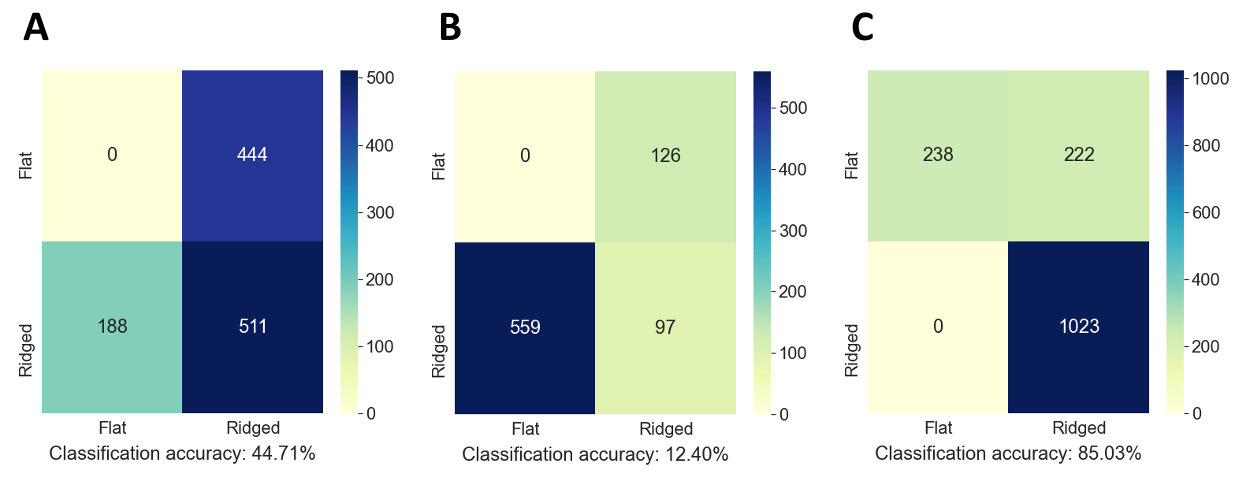

Supplement: Fig S14 — Shown are the results for training/test set splits 1–3 (panels A–C, respectively). The classification is for Dictyostelium cells moving on flat vs. ridged surfaces; the test and training sets are conditioned on the presence of the external electric field. (PNG) [file pone.0318036.s014.png]

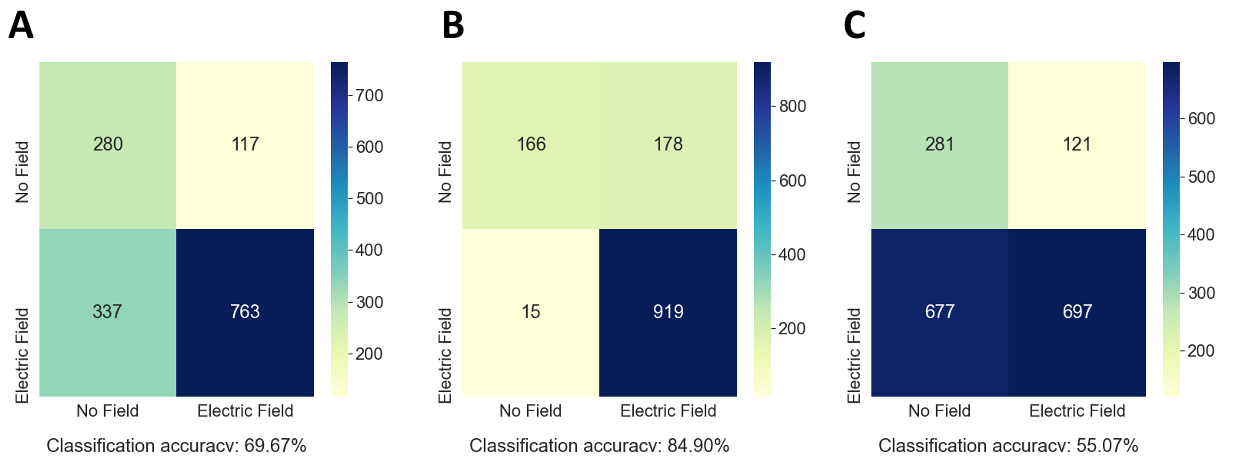

Supplement: Fig S15 — Shown are the results for training/test set splits 1–3 (panels A–C, respectively). The classification is for Dictyostelium cells moving in the presence vs. absence of the external electric field; the test and training sets are not conditioned on the nano-topography type. (PNG) [file pone.0318036.s015.png]

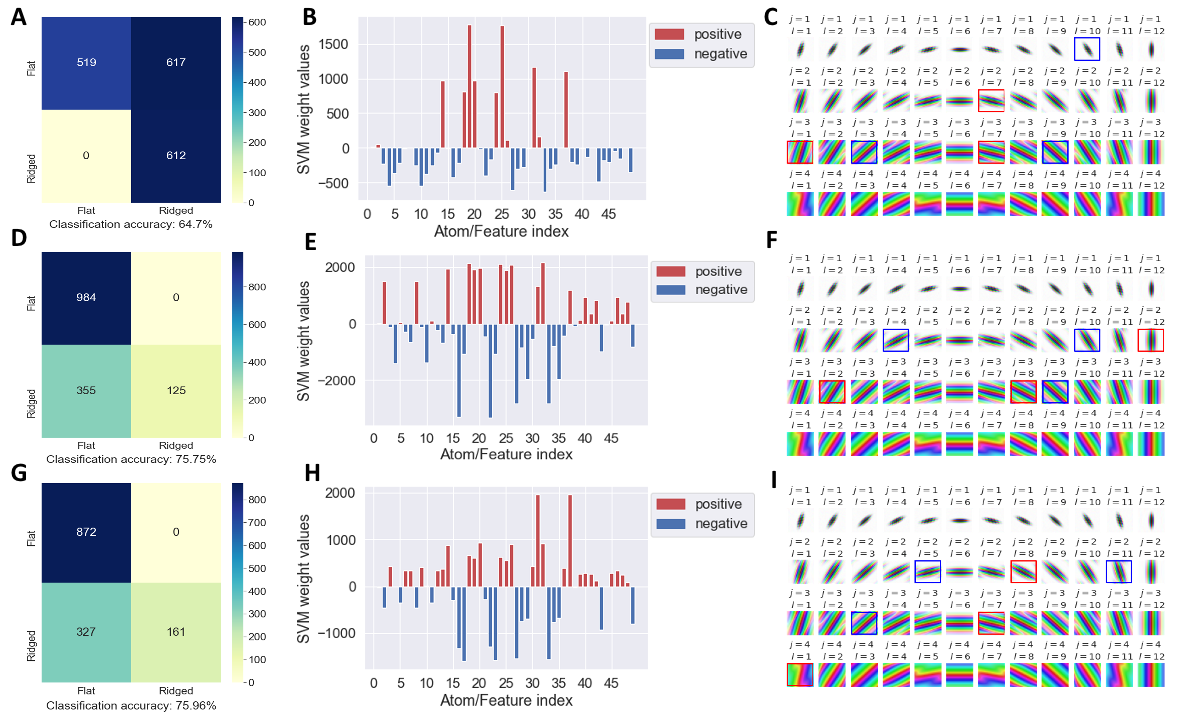

Supplement: Fig S16 — The features characterize images of Dictyostelium cells on flat vs. ridged surfaces in the absence of the external electric field. Panels A–C: training/test set split 1, panels D–F: split 2, panels G–I: split 3. Panels (A,D,G) are the confusion matrices for the SVM classification into two nano-topography types (all SVM parameters are as in the original dataset). Panels (B,E,H) show SVM weights assigned to each scattering transform; the 1–49 numbering on the x-axis corresponds to the zeroth-order transform followed by the first-order transforms in the order displayed in S12 Fig. Note that the positive/negative SVM weights describe features of cells on ridged/flat surfaces, respectively. In panels (C,F,I), the features corresponding to top 3 positive (red squares) and negative (blue squares) SVM weights are highlighted. (PNG) [file pone.0318036.s016.png]

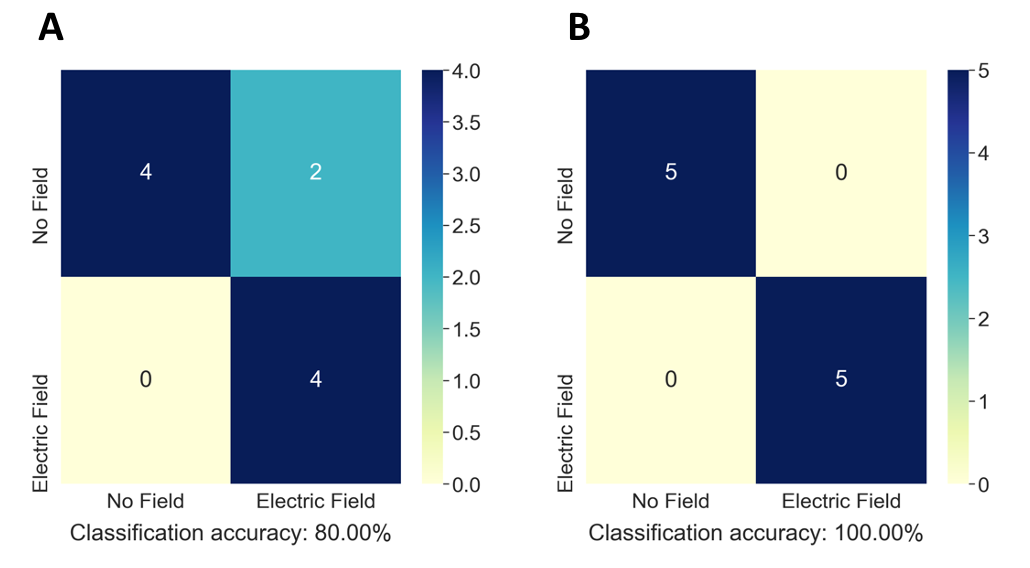

Supplement: Fig S17 — Shown are the results for random training/test set splits 2 (A) and 3 (B). The classification is for Dictyostelium cells moving in the presence vs. absence of the external electric field, regardless of the nano-topography type. (PNG) [file pone.0318036.s017.png]

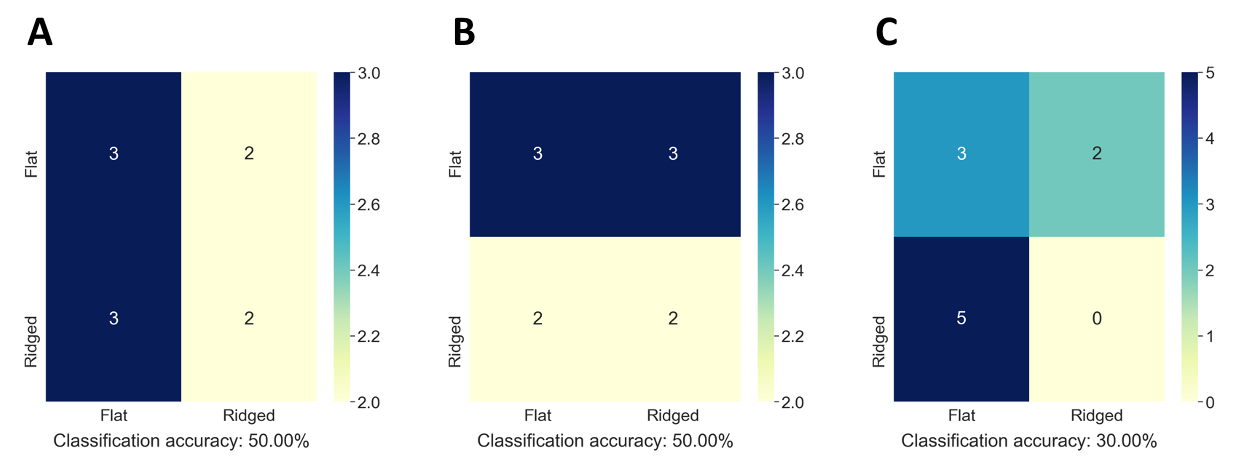

Supplement: Fig S18 — Shown are the results for random training/test set splits 1–3 (panels A–C, respectively). The classification is for Dictyostelium cells moving on flat vs. ridged surfaces; the test and training sets are not conditioned on the presence or absence of the external electric field. (PNG) [file pone.0318036.s018.png]

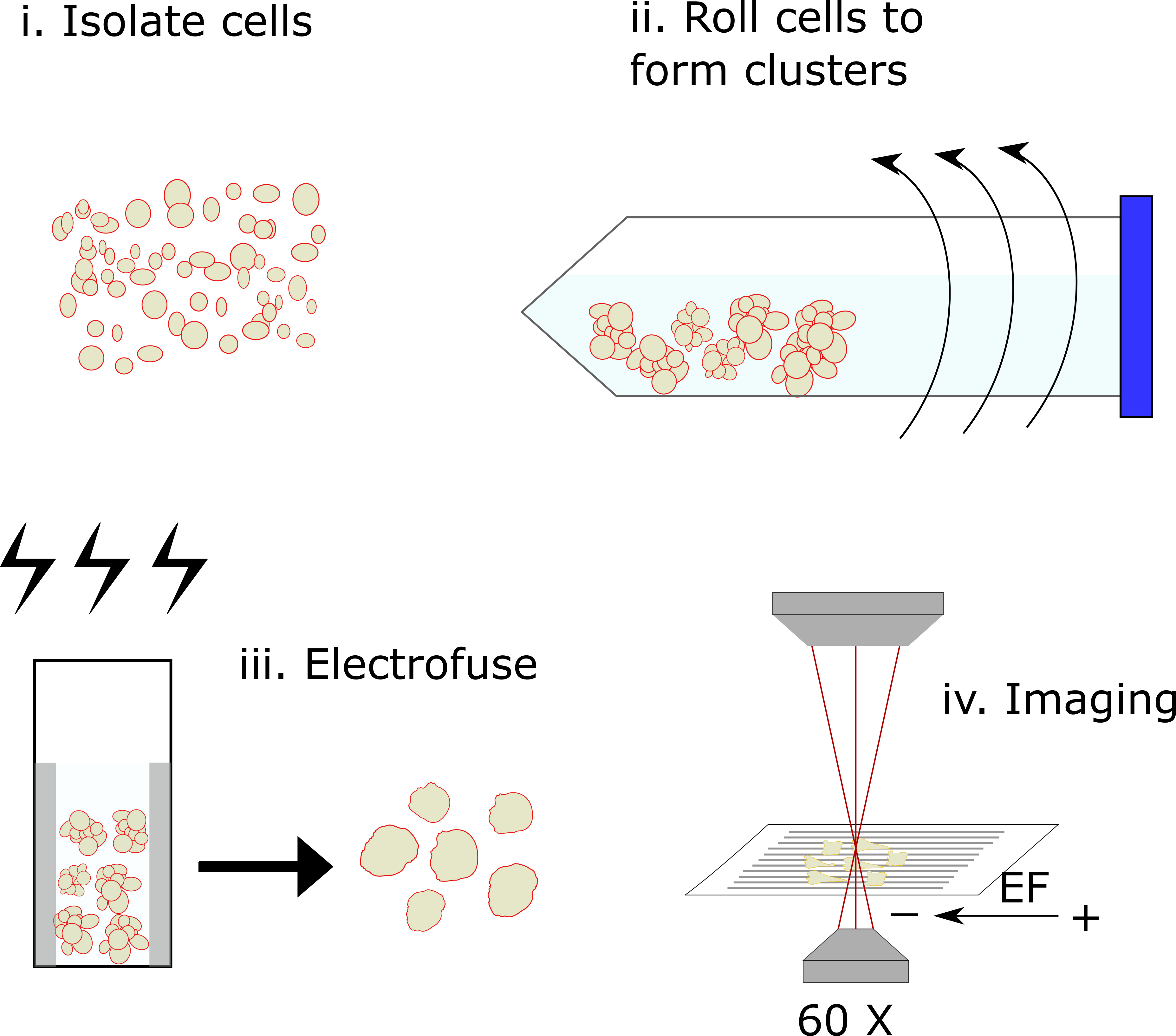

Supplement: Fig S19 — There are four steps in the experimental procedure. (i): Isolate D. discoideum cells from the cell culture. The cell media is centrifuged twice to remove the nutrients from the culture media. (ii): Cells are set in the 15 mL centrifuge tube and the tube is rolled for 30 min to cluster single cells together. (iii): After rolling, three electric pulses at the voltage of 1 kV are applied to the cells to open up the cell membranes and form giant cells by fusion. (iv): After electrofusion, the cells are transferred to the nano-ridges and incubated for 1–2 h before imaging. Afterwards, the chamber is transferred to the imaging core and the data are collected using a spinning disk confocal microscope. (PNG) [file pone.0318036.s019.png]

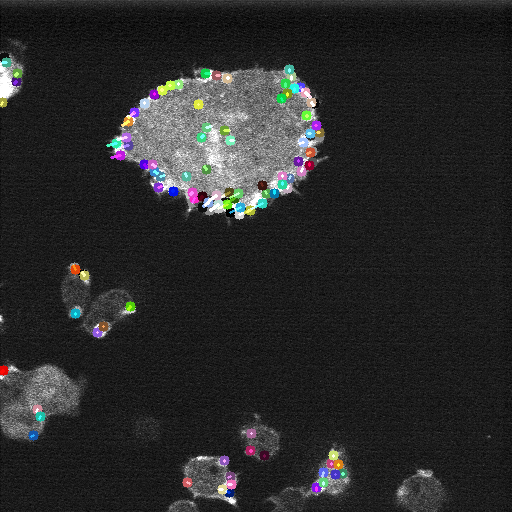

Supplement: Movie S2 — Fig 7D–7F shows the first, middle, and last frames from this video. (TIFF) [file pone.0318036.s021.tiff]
